# Correlations Between Coffee Intake, Glycemic Control, Cardiovascular Risk, and Sleep in Type 2 Diabetes and Hypertension: A 12-Month Observational Study

**DOI:** 10.3390/biomedicines13081875

**Published:** 2025-08-01

**Authors:** Tatiana Palotta Minari, José Fernando Vilela-Martin, Juan Carlos Yugar-Toledo, Luciana Pellegrini Pisani

**Affiliations:** 1 Department of Bioscience, Federal University of São Paulo (UNIFESP), Santos 11015-020, Brazil; pisani@unifesp.br; 2 Department of Hypertension, State Faculty of Medicine of São José do Rio Preto (FAMERP), São José do Rio Preto 15090-000, Brazil; vilelamartin@uol.com.br (J.F.V.-M.); yugarjuan@uol.com.br (J.C.Y.-T.)

**Keywords:** coffee consumption, glycated hemoglobin, LDL cholesterol, sleep duration, chronic diseases, women

## Abstract

**Background:** The consumption of coffee has been widely debated regarding its effects on health. This study aims to analyze the correlations between daily coffee intake and sleep, blood pressure, anthropometric measurements, and biochemical markers in individuals with type 2 diabetes (T2D) and hypertension over a 12-month period. **Methods:** An observational study was conducted with 40 participants with T2D and hypertension, comprising 20 females and 20 males. Participants were monitored for their daily coffee consumption over a 12-month period, being assessed every 3 months. Linear regression was utilized to assess interactions and relationships between variables, providing insights into potential predictive associations. Additionally, correlation analysis was performed using Pearson’s and Spearman’s tests to evaluate the strength and direction of linear and non-linear relationships. Statistical significance was set at *p* < 0.05. **Results:** Significant changes were observed in fasting blood glucose (FBG), glycated hemoglobin (HbA1c), body weight, body mass index, sleep duration, nocturnal awakenings, and waist-to-hip ratio (*p* < 0.05) over the 12-month study in both sexes. No significant differences were noted in the remaining parameters (*p* > 0.05). The coffee consumed by the participants was of the “traditional type” and contained sugar (2 g per cup) for 100% of the participants. An intake of 4.17 ± 0.360 cups per day was found at baseline and 5.41 ± 0.316 cups at 12 months (*p* > 0.05). Regarding correlation analysis, a higher coffee intake was significantly associated with shorter sleep duration in women (*r* = −0.731; *p* = 0.037). Conversely, greater coffee consumption correlated with lower LDL cholesterol (LDL-C) levels in women (*r* = −0.820; *p* = 0.044). Additionally, a longer sleep duration was linked to lower FBG (*r* = −0.841; *p* = 0.031), HbA1c (*r* = −0.831; *p* = 0.037), and LDL-C levels in women (*r* = −0.713; *p* = 0.050). No significant correlations were observed for the other parameters in both sexes (*p* > 0.05). **Conclusions:** In women, coffee consumption may negatively affect sleep duration while potentially offering beneficial effects on LDL-C levels, even when sweetened with sugar. Additionally, a longer sleep duration in women appears to be associated with improvements in FBG, HbA1c, and LDL-C. These correlations emphasize the importance of a balanced approach to coffee consumption, weighing both its potential health benefits and drawbacks in postmenopausal women. However, since this study does not establish causality, further randomized clinical trials are warranted to investigate the underlying mechanisms and long-term implications—particularly in the context of T2D and hypertension.

## 1. Introduction

Coffee is a globally cherished beverage, valued for its rich flavor, invigorating aroma, and stimulating effects, primarily driven by its caffeine content [1]. Beyond its sensory appeal, coffee holds deep social and cultural significance, often serving as a communal staple worldwide [2,3]. Brazil plays a pivotal role in the global coffee market, solidifying its position as the world’s largest producer, with approximately 54.2 million 60 kg bags produced in 2024 [4,5,6]. The country is also a leading exporter, having shipped 50.5 million bags, generating USD 12.3 billion in revenue—a 28.8% increase from the previous year [3]. The primary destinations for Brazilian coffee include the United States, Germany, Italy, Belgium, and Japan [5,6]. Given its extensive global presence and integral role in daily life, coffee has naturally become a subject of significant scientific inquiry, with researchers examining its diverse effects on human health [7,8,9].

Numerous epidemiological studies suggest that moderate coffee consumption is associated with a lower risk of various chronic diseases, including cardiovascular conditions, type 2 diabetes (T2D), and hypertension [7,8,9,10,11,12]. Bioactive compounds such as chlorogenic acids, diterpenes (cafestol and kahweol), and caffeic acid contribute to its antioxidant and anti-inflammatory properties, potentially offering protective effects [12,13,14,15]. On the other hand, some research warns of the risks of excessive consumption, citing potential increases in blood pressure and heart rate that could impact cardiovascular health [16,17,18]. Additionally, coffee’s influence on sleep patterns has been widely debated, with caffeine acting as a central nervous system stimulant that can disrupt the sleep architecture by delaying onset, increasing nocturnal awakenings, and reducing overall sleep quality, especially when consumed in the afternoon or evening [18,19,20,21,22,23,24,25]. Individual variability in caffeine metabolism further complicates this relationship, as some people experience prolonged stimulant effects that interfere with sleep, while others metabolize caffeine more efficiently, mitigating its impact on sleep disturbances [25,26,27,28,29,30].

Considering the conflicting findings on coffee consumption and health, further research is essential to clarify its physiological effects. This study aims to evaluate the correlations between coffee intake and glycated hemoglobin (HbA1c), fasting blood glucose (FBG), total cholesterol (TC), low-density lipoprotein cholesterol (LDL-C), high-density lipoprotein cholesterol (HDL-C), serum triglycerides (TGs), body weight, body mass index (BMI), waist circumference (WC), waist-to-hip ratio (WHR), blood pressure (BP), heart rate, and sleep parameters—including nocturnal awakenings, sleep duration, and difficulty falling asleep—in patients with T2D and hypertension over a 12-month period.

## 2. Materials and Methods

### 2.1. Study Design

This research followed a 12-month observational approach between 2023 and 2024, incorporating insights from the fifth evaluation of a dataset derived from prior studies. The data and patient sample were drawn from records linked to the outpatient care system of the hospital FUNFARME, which is located in São José do Rio Preto and connected to the state medical school (FAMERP). Throughout this study, data were systematically gathered and examined quarterly to track developments and identify emerging patterns.

### 2.2. Sample Size

A power calculation was conducted to determine the appropriate sample size required to detect an effect size (f^2^) of 0.10, with a significance level (α) of 0.05 and a statistical power of 0.80 for a two-tailed test. The analysis assessed the necessity of a 40 participant sample and evaluated whether sex-specific statistical analysis—with 22 men and 22 women—provided adequate statistical power and reliability. The sample size and effect size calculations were based on the methodology outlined in a previous study. The primary dependent variable selected was glycated hemoglobin. To further ensure sample size adequacy, a post hoc power analysis was conducted, confirming the originally estimated statistical power of 0.80. The output parameters included δ (1.0050378), degrees of freedom (98), and a critical *t*-value (1.9844675). The achieved statistical power was 0.79, indicating a high probability of detecting a true effect, thereby validating the methodological integrity of the initial sample size calculation. Additionally, an estimated dropout rate of 10% was accounted for over the 12-month study period, out of a total of 44 participants. Consequently, a minimum of 40 individuals were required to complete the project without compromising statistical validity. The calculation was performed using the G*Power 3.1.9.6^®^ software [computer software: Schleswig-Holstein, Germany] [31].

### 2.3. Inclusion Criteria

Participants were aged between 18 and 80 years and had a confirmed diagnosis of both T2D and hypertension. T2D was defined by FBG levels ≥ 126 mg/dL and HbA1c levels ≥ 6.5%. Hypertension was diagnosed based on persistent systolic and diastolic blood pressure readings ≥ 140/90 mmHg, respectively. Both male and female participants were included to ensure sample diversity. Inclusion criteria also required a BMI of ≥30.0 kg/m^2^, regular coffee consumption (minimum one cup daily), availability for quarterly consultations over 12 months, willingness to provide informed consent, and full participation in study procedures. Patients undergoing other dietary interventions were excluded.

### 2.4. Exclusion Criteria

Individuals were excluded from the study if they had difficulty completing study instruments, demonstrated impediments to regular data collection, or lacked a confirmed diagnosis of both T2D and hypertension. Exclusion also applied to those undergoing insulin therapy, using sodium–glucose cotransporter-2 (SGLT-2) inhibitors, or glucagon-like peptide-1 (GLP-1) analogs, as these medications can significantly alter metabolic outcomes and potentially confound intervention effects [32].

Participants diagnosed with chronic kidney disease (defined as a creatinine clearance of <60 mL/min/1.73 m^2^), those considered eutrophic or malnourished (BMI < 18.5 kg/m^2^), and those who practiced physical exercise for more than 150 min a week were also excluded. Additional exclusion criteria included ongoing nutritional monitoring, adherence to weight loss diets, active liver disease, cancer or similar conditions under treatment, pregnancy or lactation, a history of caffeine sensitivity or coffee allergy, and the use of medications that could interfere with study variables (e.g., sleep aids and antidepressants). Moreover, individuals who consumed decaffeinated coffee or did not consume coffee at all were not eligible for participation.

### 2.5. Flowchart Sample Selection

**Figure 1:**
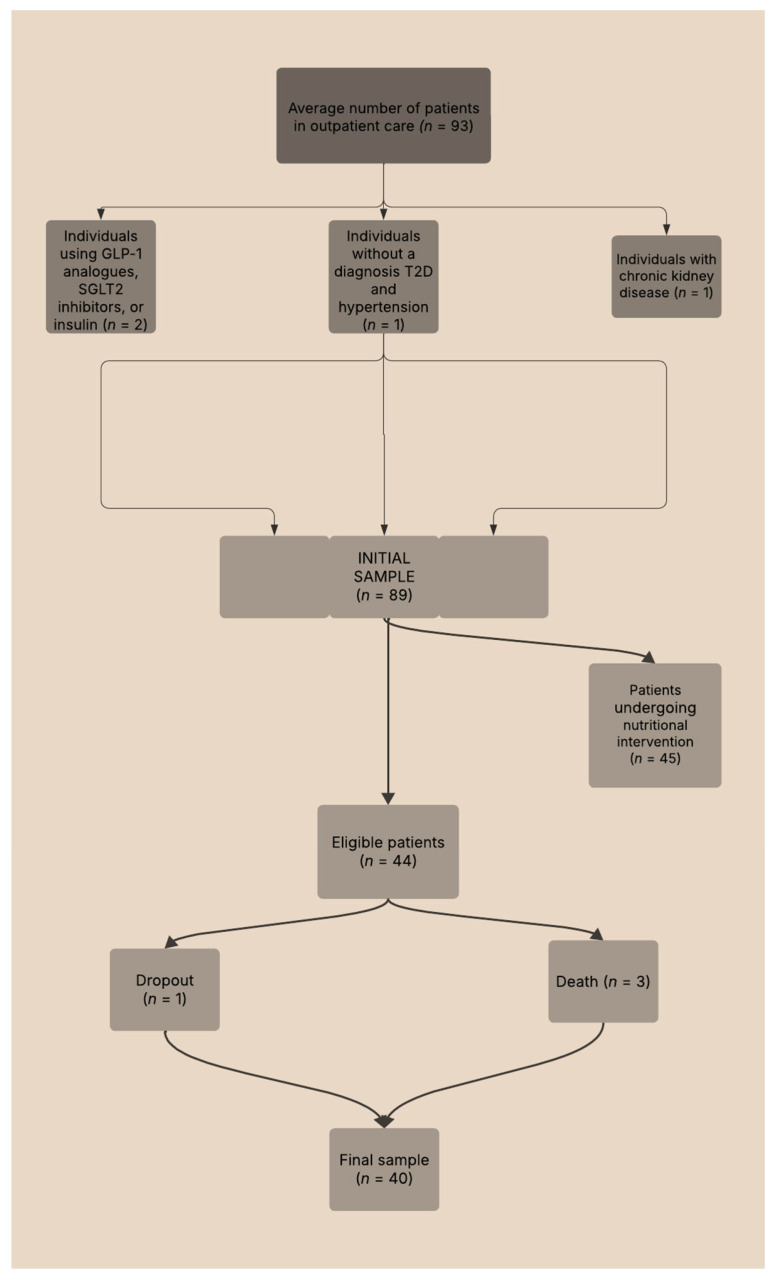
Flowchart of the sample selection process for study participants.

In the original study, 93 individuals were recruited from a convenience sample—45 underwent a nutritional intervention, while 44 formed the control group. For this study, 44 participants were selected as they had not received any nutritional intervention. Due to complications, including participant withdrawals and fatalities throughout the project, the final sample size was reduced to 40, evenly distributed between 20 women and 20 men. Figure 1 presents an overview of the participant selection methodology utilized throughout the research.

### 2.6. Data Collection Procedures 

#### 2.6.1. Laboratory Measurements

Blood samples were collected and examined at the Clinical Analysis Laboratory affiliated with the hospital. A certified nursing technician carried out the venipuncture procedure after participants underwent a fasting period of up to 12 h. The laboratory assessments for HbA1c and blood glucose concentrations were performed using diagnostic instruments manufactured by Siemens^®^ (Berlin, Germany). The diagnostic parameters for T2D were defined as either two separate readings of FBG levels at or above 126 mg/dL following a fasting period of no less than 8 h, or HbA1c levels equal to or exceeding 6.5% [32].

The assessment of TC, TG, and HDL-C was conducted using the Roche cobas c501 analyzer (Basel, Switzerland). LDL-C values were determined via the Friedewald formula: LDL-C = TC − HDL-C − (TG ÷ 5). In instances where triglyceride concentrations exceeded 400 mg/dL, LDL-C was instead measured directly with the Roche cobas c501^®^ system, employing Sekisu^®^ reagents (Beijing, China). According to reference standards, LDL-C should be maintained below 130 mg/dL in healthy individuals, while those with cardiovascular risks are advised to target levels under 70 mg/dL. HDL-C is considered optimal when above 40 mg/dL. For TC, values below 190 mg/dL are deemed normal, while those between 200 and 239 mg/dL are categorized as borderline, and levels above 240 mg/dL indicate hypercholesterolemia. Ideally, TG levels should remain under 130 mg/dL [32].

#### 2.6.2. Blood Pressure Measurements

BP assessments were carried out using Omron Control Digital^®^ monitors (Rio de Janeiro, Brazil), with participants resting between 5 and 10 min before the procedure. Three measurements were recorded on both the right and left upper arms, spaced by one-minute intervals. For cases presenting initial readings above 160/90 mmHg, an additional rest period of five minutes was observed before re-evaluation. When values exceeded 200/100 mmHg, and the digital equipment failed to obtain reliable readings, a manual evaluation was conducted using a Nylon Premium Black^®^ sphygmomanometer and a Solidor CX^®^ dual-head stethoscope (both manufactured in São Paulo, Brazil). Individuals exhibiting a systolic pressure ≥ 140 mmHg and/or a diastolic pressure ≥ 90 mmHg were classified as hypertensive [33].

#### 2.6.3. Anthropometric Measurements

Anthropometric evaluations were carried out using precision equipment, including the Welmy W200a^®^ digital scale and an integrated stadiometer (São Paulo, Brazil), to record body weight in kilograms and height in centimeters. Participants arrived wearing light clothing and were instructed to avoid heavy meals beforehand. Footwear was removed, and bladder emptying was recommended before measurement. Individuals stood upright against the device’s vertical rod, maintaining a straight posture while facing forward. Data collection occurred in the early morning hours to reduce variability in hydration and body weight caused by daily physiological fluctuations.

The body mass index (BMI) was computed as the ratio of weight to squared height (kg/m^2^), enabling classification into internationally recognized categories: underweight (<18.5), normal weight (18.5–24.9), overweight (25.0–29.9), and obesity grades I (30.0–34.9), II (35.0–39.9), and III (≥40.0). For older adults (60+ years), adapted ranges were applied, identifying underweight (≤22.0), normal (22.0–27.0), and obesity (≥27.0) [32,33].

Measurements of waist and hip circumferences were made using a Sanny^®^ flexible tape (2.0 m, São Paulo, Brazil). Waist size was taken midway between the lowest rib and the iliac crest, marked beforehand to ensure consistency. Reference thresholds classified the cardiovascular risk as low (<80 cm for women and <94 cm for men), moderate (80–87 cm for women and 94–101 cm for men), and high (>88 cm for women and >102 cm for men). The waist-to-hip ratio, indicating fat distribution, was calculated by dividing the waist measurement by the hip measurement, which was taken at the widest portion of the hips and buttocks. Ratios below 0.85 for women and below 0.9 for men reflected a low cardiovascular risk; values exceeding these levels indicated an elevated risk [33]. All data mentioned in this section were evaluated every 3 months over 12 months.

#### 2.6.4. Sleep Quality and Duration Assessments

During the traditional consultation, the Pittsburgh Sleep Quality Index (PSQI) was utilized, which included questions such as: ‘What is the duration of your sleep (in hours)?’, ‘Do you have difficulty falling asleep (yes/no)?’, and ‘Do you wake up during the night (yes/no)? If so, how many times do you wake up (quantity/number)?’ Furthermore, the questionnaire comprises seven components that analyze different aspects of sleep: subjective sleep quality, sleep latency, sleep duration, habitual sleep efficiency, sleep disturbances, the use of sleeping medication, and daytime dysfunction. Each component is scored from 0 to 3, with higher scores indicating poorer sleep quality. The total score ranges from 0 to 21, with a score above 5 suggesting poor sleep quality. The PSQI is a valuable tool in both clinical and research settings, providing a comprehensive understanding of an individual’s sleep patterns and disturbances. Validated across various populations, the PSQI is essential for identifying sleep-related problems and monitoring therapeutic interventions. This questionnaire demonstrated a Cronbach’s alpha of 0.83 [34]. All data mentioned in this section were evaluated quarterly over 12 months.

#### 2.6.5. Coffee Consumption

During consultation, both a food frequency questionnaire (FFQ) [35,36] and a habitual dietary recall were used to assess coffee consumption patterns and overall dietary habits. The FFQ included questions such as: ‘How many cups of coffee do you drink daily (quantity/number)?’, ‘Do you drink your coffee with or without sugar (yes/no)?’, and ‘What type of coffee do you consume: traditional, specialty, or gourmet? (types)’. Additional questions covered ‘Which coffee brand do you consume (brand)?’ and ‘What is your preparation method—how many tablespoons of coffee per liter of water (quantity/number)?’ and ‘Coffee intake time: Before 15 h or after 16 h?’. The habitual dietary recall complemented the FFQ by gathering more detailed and personalized information about the participants’ regular food intake, meal composition, and nutrient distribution over time. This methodology allowed for a broader understanding of dietary patterns beyond coffee consumption, ensuring a more comprehensive analysis of its possible interactions with other lifestyle factors. The validation of Cronbach’s alpha for the FFQ was 0.70 [36]. All data mentioned in this section were evaluated every 3 months over the course of 12 months. Note: Each cup of coffee was 50 mL.

#### 2.6.6. Medications, Exercise, and Diet

All participants were treated with oral hypoglycemic agents, including metformin and/or gliclazide, and maintained consistent medication regimens throughout the study period. Additional documented drugs included antihypertensives (e.g., hydrochlorothiazide, furosemide, losartan, captopril, enalapril, atenolol, amlodipine, and ramipril) and lipid-lowering therapy with simvastatin (40 mg). These medications were primarily prescribed during consultations at the Hypertension or Endocrinology Outpatient Clinics and were available free of charge at Unified Health System (SUS) pharmacies. There were no changes in medications or doses throughout the study. 

Participants were inactive at the study’s outset and remained sedentary throughout the research period, only engaging in routine daily activities such as house cleaning, grocery shopping, and dog walking. High consumption of ultra-processed foods, which are rich in refined carbohydrates and saturated fats (such as cakes, sweets, cookies, snacks, soft drinks, delivery meals, frozen foods, hamburgers, etc.), was observed over 12 months. No changes in eating patterns or caloric intake were observed. It is important to note that the patients’ diet was slightly hypercaloric (total energy value > total energy expenditure) both at the beginning and at the end of the study. 

Additionally, comprehensive data from the analysis of the patients’ dietary nutritional composition is available in Table S1 (general) and Table S2 (separated by sex) of the Supplementary Materials Section for this study. All data on medication, exercise, and diets were collected during consultations using the clinical evaluation protocol throughout the intervention from the 1st to the 12th month. The Cronbach’s alpha of this instrument is 0.91 [35]. These data were collected to control for any type of bias during the study. All data mentioned in this section were evaluated every 3 months over 12 months.

#### 2.6.7. Analysis Period

The total duration of the study was 12 months, during which data on coffee consumption, laboratory measurements, anthropometric measurements, and sleep quality and duration were collected and analyzed every three months to assess changes and trends over the study period. Linear regression and correlation analyses were performed at baseline and subsequently at 12 months.

#### 2.6.8. Ethical Aspects

This study was conducted in strict accordance with the principles of the Declaration of Helsinki and received approval from the Institutional Ethics Committee of the FAMERP, specifically from the Human Research Ethics Committee (CAAE: 33554520.0.0000.5415). The approval was granted on 18 July 2020. This research was registered in Clinical Trials (Registry number: NCT06235762).

Informed consent was obtained from all participants in the study. The patients provided their written informed consent for the publication of this paper. Steps were taken to ensure their confidentiality and anonymity, thereby safeguarding the identities of the interviewees.

#### 2.6.9. Data Availability Statement

The data were gathered and managed using the REDCap 14.0.9 electronic data capture tools^®^, which are hosted at REDCap—FUNFARME/FAMERP [37]. The data will be made available upon request to the corresponding author.

#### 2.6.10. Statistics

Descriptive statistics were used to summarize the data. For parameters that followed a normal distribution (parametric data), the mean and standard deviation (SD) were presented. In contrast, for parameters that did not follow a normal distribution (non-parametric data), the median and interquartile range (IQR) were reported. 

To assess changes in health variables and coffee consumption over time, the dependent variables underwent normality and sphericity tests, including the Shapiro–Wilk and Mauchly tests, respectively. A Repeated measures ANOVA was performed to examine differences in parametric health parameters across four time points (baseline, 3 months, 6 months, 9 months, and 12 months) within the same participants. Tukey’s post hoc test was applied to identify specific differences. For non-parametric markers, the Friedman test was utilized, followed by the Durbin–Conover post hoc test. Additionally, the analysis was conducted separately for each sex to investigate potential sex-based differences in the effects of coffee consumption on health and sleep parameters.

In the tables illustrated in the Supplementary Materials Section, data with a Gaussian distribution were presented as the mean ± the standard deviation (SD), while data with a non-Gaussian distribution were presented as the median ± the interquartile range (IQR). In the graphs illustrated in the manuscript, data were presented with confidence intervals. Confidence intervals that do not intersect represent significant differences.

Statistical analyses were performed to examine the relationship between daily coffee intake (independent variable) and changes in health and sleep-related outcomes (dependent variables). Linear regression tests were applied at baseline and at 12 months to assess predictive strength. The coefficient of determination (R^2^) was used to quantify how well coffee consumption predicted changes in variables such as BP, FBG, HbA1c, BMI, total sleep time, and sleep efficiency. Higher R^2^ values indicated stronger predictive power [38].

Pearson correlation coefficients (*r*) evaluated the direction and magnitude of linear associations between coffee consumption and the same set of continuous health and sleep variables. Strong correlations were defined as *r* > 0.7, moderate as 0.3–0.7, and weak as 0.1–0.3. For variables that did not show clear linear trends, Spearman’s rank correlation was used to detect potential non-linear associations [39]. These analyses helped identify which outcomes were most closely associated with coffee intake over time.

For all calculations, α = 0.05 and *p* < 0.05 were adopted. The statistical analysis for this study was conducted using the Jamovi project (Jamovi (Version 2.6.23) ^®^ [computer software: Sydney, Australia]; retrieved from https://www.jamovi.org) [40].

## 3. Results

### 3.1. Description of Demographic Data

Table 1 presents the sociodemographic data collected at baseline, providing an overview of participant characteristics at the start of the study.

### 3.2. Description of Data Collected

Table 2 presents the comparison between the baseline and the 12-month mark, showcasing anthropometric, laboratory, and sleep data. A more comprehensive analysis, conducted every 3 months throughout the 12 months, is illustrated in the Supplementary Material Section through Figures S1–S5. Additionally, out of sheer curiosity, a comparative table between the baseline and the 12th month, without sex differentiation, has also been provided in the Supplementary Materials Section (Table S3).

Over the 12-month study, several parameters exhibited statistically significant changes. Both women and men showed a notable increase in FBG and HbA1c levels, as well as a significant rise in body weight and BMI. Sleep duration declined considerably, accompanied by an increase in nocturnal awakenings. Additionally, the waist-to-hip ratio increased significantly. These findings highlight substantial alterations in glycemic control, body weight, sleep patterns, and fat distribution over time. No significant differences were observed in the remaining parameters or between sexes.

**Figure 2:**
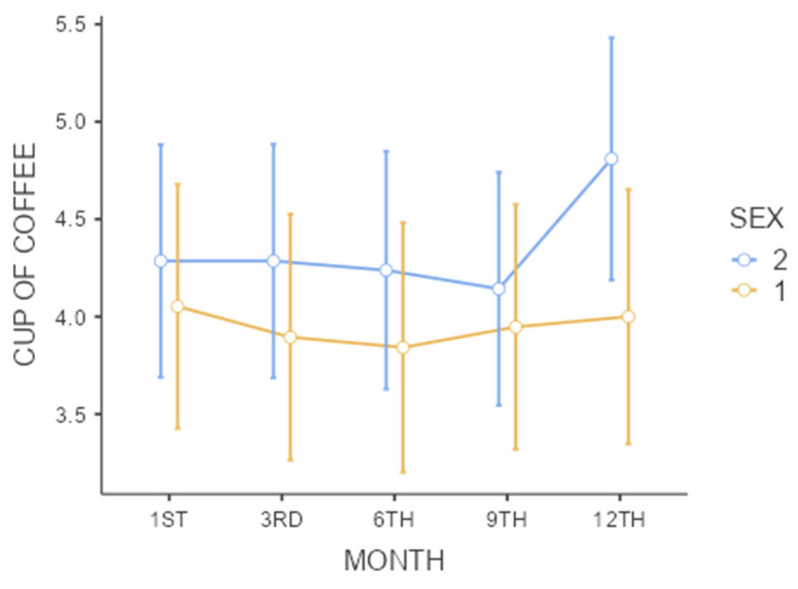
Coffee intake (cups) over 12 months. Normal data are expressed as the mean ± the standard deviation (SD). Confidence intervals that do not intersect represent significant differences (a repeated measures ANOVA followed by Tukey’s post hoc test for parametric data); Sex: 1—men and 2—women; no significant differences were found in the time period studied within each sex or between sexes. * *p* < 0.05. Note: One cup = 50 mL.

Table 3 illustrates the coffee consumption patterns of the participants over 12 months, including the number of cups consumed, types of coffee, and sweetening preferences. The data are categorized by gender and presented with baseline and 12-month values, highlighting no significant changes in consumption habits. Subsequently, coffee consumption trends are presented over 12 months in Figure 2.

Table 4 highlights the diversity in coffee brand preferences and preparation methods among participants, as well as the consistency in caffeine content for the major brands. This diversity reflects the varied tastes and habits of the participants while maintaining a uniform caffeine content across popular brands such as 3 Corações^®^ (Eusébio, Ceará, Brazil), Pilão^®^ (Natal, Rio Grande do Norte, Brazil), and Melitta (Varginha, Minas Gerais, Brazil); ^®^.

### 3.3. Pearson Correlation Analysis

**Figure 3:**
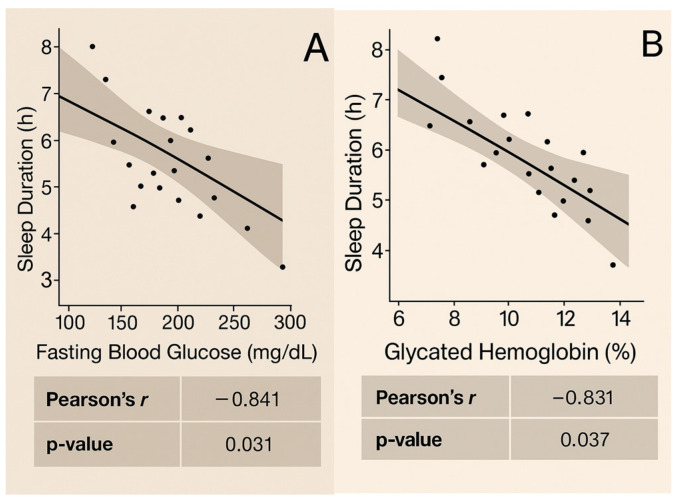

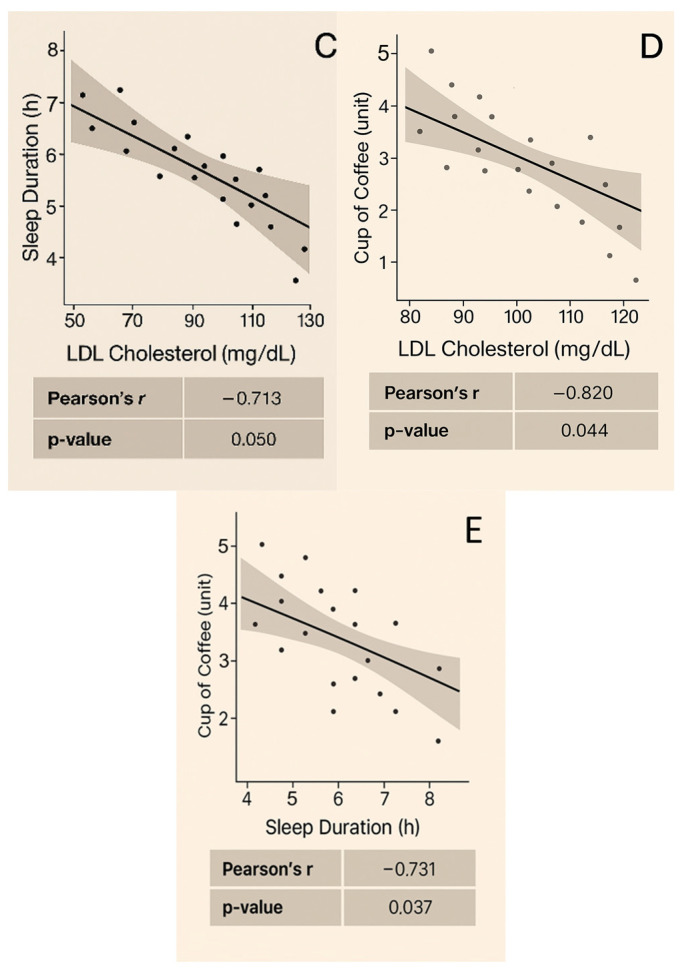
Correlations observed at baseline and at the 12th month in women: (**A**) Sleep duration shows a strong inverse correlation with FBG at baseline (*r* = −0.841, *p* = 0.031). (**B**) Sleep duration shows a strong inverse correlation with HbA1c at baseline (*r* = −0.831, *p* = 0.037). (**C**) Sleep duration shows an inverse correlation with LDL cholesterol at baseline (*r* = −0.713, *p* = 0.050). (**D**) Coffee consumption shows a strong inverse correlation with LDL-C at baseline (*r* = −0.820, *p* = 0.044). (**E**) Sleep duration shows a strong inverse correlation with coffee consumption at the 12-month mark (*r* = −0.731, *p* = 0.037).

Correlation analysis was conducted only at baseline and 12 months for the parameters related to glycemic control, cardiovascular risk, and sleep, namely FBG, HbA1c, TC, HDL-C, LDL-C, TG, weight, BMI, WHR, waist circumference, BP, heart rate, sleep duration, and nocturnal awakenings, in both sexes. Figure 3 presents the graphs depicting significant correlation results in women (*p* < 0.05). No strong correlations were found for men (*p* > 0.05).

### 3.4. Linear Regression Analysis and Spearman’s Test

Variables exhibiting strong correlations (*r* > 0.7 or *r* > −0.7) in the Pearson analysis also demonstrated affinity, relation, and interaction in the linear regression (*p* < 0.05). Conversely, variables with very weak correlations (*r* close to 0 or *r* < 0.3 or *r* < −0.3) did not yield significant results in the linear regression (*p* > 0.05). For these variables, the Spearman test was applied to account for non-linear data, which similarly indicated a low-strength relationship for all analyzed data. These variables possess a very low correlation strength [*ρ* (rho) close to zero, *ρ* < 0.3, or *ρ* < −0.3, *p* > 0.05]. 

The complete and detailed information, including correlations and relationships ranging from weak to strong, separated by sex at both baseline and the 12-month mark, can be found in the Supplementary Materials Section from Table S4 to Table S7.

## 4. Discussion

The findings of this study suggest a multifaceted relationship between coffee consumption and various health parameters in women. Daily coffee consumption ranging from 4.17 ± 0.360 to 5.41 ± 0.316 cups per day (equivalent to 208.5 mL to 270.5 mL of coffee daily, based on a serving size of a 50 mL per cup) was significantly associated with reduced sleep duration. This intake exceeds the commonly recommended limit of 3 to 4 cups per day, as advised in the current literature to help mitigate potential negative impacts on sleep and overall health [41,42,43,44,45]. Given that each 50 mL cup contains approximately 95–120 mg of caffeine, this corresponds to a daily intake of 396.15 to 649.2 mg of caffeine—substantially surpassing the suggested upper limit of 400 mg/day for most adults [11,12,13]. These findings underscore the critical need to moderate caffeine intake, particularly among individuals susceptible to sleep disturbances. Excessive consumption may impair sleep quality due to caffeine’s stimulant properties, which can disrupt the body’s natural circadian rhythm and interfere with the ability to achieve restful sleep [45]. Conversely, coffee consumption may demonstrate beneficial effects on metabolic health, as observed in recent studies [41,42]. 

Several epidemiological studies indicate that moderate coffee consumption is associated with improved endocrine function, particularly in lipid regulation and insulin sensitivity [41,42,43]. The observed reductions in LDL-C align with findings from meta-analyses and large cohort studies, emphasizing coffee’s role in modulating cholesterol metabolism and enhancing glucose homeostasis, especially in women [7,44,45,46]. These beneficial effects are primarily attributed to diterpenes such as cafestol and kahweol, which influence multiple metabolic pathways. By inhibiting intestinal cholesterol absorption, these compounds lower circulating LDL-C levels [44] while simultaneously regulating the expression of key enzymes in hepatic cholesterol synthesis, such as cholesterol 7-alpha-hydroxylase (CYP7A1) and sterol regulatory element-binding protein 2 (SREBP-2) [7,43,44,45,46]. Additionally, diterpenes activate peroxisome proliferator-activated receptor alpha (PPARα), enhancing fatty acid oxidation and contributing to a more favorable lipid balance [47].

Coffee consumption has also been linked to increased HDL-C levels, which facilitate cholesterol transport from the arteries to the liver for metabolism and excretion, thereby reducing atherosclerotic plaque formation [48]. Diterpenes promote the activity of proteins and enzymes responsible for HDL synthesis and transport, improving overall lipid profiles. Studies suggest that these bioactive compounds inhibit 3-hydroxy-3-methylglutaryl-CoA (HMG-CoA) reductase, a key enzyme in cholesterol biosynthesis, leading to reduced hepatic cholesterol production. Additionally, they enhance the expression of LDL receptors, improving cholesterol clearance and further lowering circulating LDL-C levels. Cafestol and kahweol also modulate fatty acid and triglyceride synthesis, reducing visceral fat accumulation and contributing to broader metabolic benefits. These processes are more pronounced in women compared to men [48,49,50].

Moreover, chlorogenic acid, another bioactive component of coffee, plays a crucial role in lipid metabolism by facilitating LDL receptor expression, thereby accelerating cholesterol uptake and clearance from circulation [15,22]. Coffee’s antioxidant and anti-inflammatory properties further support metabolic health by suppressing pro-inflammatory mediators such as TNF-α and IL-6, both of which are linked to impaired lipid metabolism and increased cardiovascular risk [47,48,49,50,51]. Additionally, its role in improving insulin sensitivity through GLUT4 activation [30] and the inhibition of hepatic glucose production indirectly aids lipid regulation by lowering triglyceride concentrations, which often accompany elevated LDL-C expression [30,48].

Another interesting fact is that all coffees are sweetened with sugar and were of the "traditional type”. Even when sweetened and with the lower quality of the coffee type, coffee demonstrated a strong correlation with cardiovascular risk markers (LDL-C), suggesting that the chemical composition of coffee may overcome the negative effects of sugar in small amounts. The low cost of “traditional coffee” ends up being more accessible to the C and D class populations (as verified in this study). Additionally, sugar consumption is also widely observed in lower-income classes due to its low price and high caloric content, as some patients are or have been through moments of food and social vulnerability [3,4,5]. Growing evidence continues to highlight the long-term inflammatory risks of sugar intake, especially among patients with chronic noncommunicable diseases [2]. However, it is important to consider that some of the observed effects on lipid profiles may also be attributable to statin use, such as simvastatin, which is commonly prescribed to individuals with dyslipidemia [2].

Wang et al. (2025) have identified distinct patterns in coffee drinking timing and their associations with mortality rates among US adults. Their study found that drinking coffee predominantly in the morning was significantly associated with lower all-cause and cardiovascular disease-specific mortality risks. Specifically, individuals with a morning coffee drinking pattern exhibited a 16% reduction in all-cause mortality risk and a 31% reduction in cardiovascular mortality risk compared to non-coffee drinkers. This suggests that the timing of coffee consumption may play a crucial role in its health benefits, emphasizing the potential importance of morning coffee drinking as a protective factor against mortality [41]. However, they did not specifically address sex differences or the timing of coffee consumption, highlighting the need for further research, as this topic remains relatively unexplored. In our research, all participants consumed coffee until 3 PM. It is important to note that caffeine has a half-life of six hours and can affect sleep if consumed later in the day. In this sense, it should be noted that the analysis of coffee consumption must be holistic, considering the time at which it is consumed, its distribution throughout the day, the type of coffee, whether it is sweetened or not, and other relevant factors.

An interesting finding observed in this study was that the markers of FBG, HbA1c, weight, BMI, and WHR increased significantly between baseline and 12 months in both sexes, but this was probably not due to coffee, even though it was sweetened. Considering that the participants consumed approximately 4–5 cups of coffee per day and sweetened it with approximately 2 g of sugar per cup, averaging 10 g of sugar per day, which equates to 10 g of carbohydrates per day—a relatively low dose of carbohydrates (equivalent to only 8% of the average daily carbohydrate intake for a patient with T2D, which should not exceed 120 g). Therefore, small/moderate amounts may be tolerated and may not be enough to nullify the positive effects of coffee, but caution is needed in generalizing this information, especially for patients with T2D and hypertension.

There was also a high intake of ultra-processed foods (cakes, sweets, cookies, and snacks) from baseline to the 12th month. On average, patients consumed 249 g of carbohydrates at the beginning of the study (range: 249–260 g), significantly increasing to 255 g (range: 249–265 g) by the 12th month. This is a substantial amount, far exceeding the recommended intake. It is worth noting that women’s diets contained greater amounts of carbohydrates and fats than men. The patients’ diet was already slightly hypercaloric at baseline, remaining so until the 12th month, with no significant difference, but still sufficient to contribute to weight gain in the long run. Therefore, it is assumed that the diet may also have contributed to worsening some markers, and sugar-sweetened coffee is probably not related to this. It is important to emphasize that the authors of this study do not recommend the consumption of sweetened beverages. Traditionally, coffee should be consumed black, without sugar, to appreciate the notes, flavors, and aromas. However, given the results, certain small flexibilities may assist in nutritional management, thereby facilitating patient adherence to a dietary intervention. Nonetheless, individualization of the prescription is necessary.

On the other hand, the adverse effects of coffee on sleep quality corroborate previous research indicating that caffeine can delay sleep onset, reduce total sleep time, and fragment sleep architecture, especially in women [20,21,46]. These sleep disturbances are particularly pronounced when caffeine is consumed in the afternoon or evening, as it can interfere with the body’s circadian rhythms [17]. Caffeine acts as an antagonist of adenosine receptors in the brain, blocking the action of adenosine, a neuromodulator that promotes sleep. This interference prevents the onset of sleep [16,17,18]. Excessive consumption can lead to adenosine resistance. When caffeine blocks adenosine receptors, the body may create more receptors to compensate, leading to increased sensitivity and difficulty sleeping. It can also delay the circadian rhythm and reduce the production of melatonin, the hormone that regulates sleep, which leads to decreased sleep quality. Furthermore, caffeine diminishes the activity of slow waves (delta waves) crucial for deep sleep, thereby reducing the time spent in the most restorative phases of the sleep cycle [16]. These combined effects result in lower sleep efficiency, making it difficult to fall asleep, causing frequent awakenings during the night, and leading to a feeling of inadequate sleep upon waking [16,17,18,19,20].

Additionally, slow caffeine metabolizers have a variation in the enzyme cytochrome P450 1A2 (CYP1A2), which is responsible for breaking down caffeine in the body. This variation causes them to process caffeine more slowly, meaning the substance stays in their body longer, thus increasing the likelihood of side effects such as insomnia, anxiety, and palpitations [16,17]. In contrast, fast metabolizers process caffeine more quickly, reducing the likelihood of these effects. The adverse effects on sleep quality suggest that public health guidelines should also address the timing of coffee intake to minimize sleep disturbances [16]. Education on the impact of caffeine on sleep hygiene could help individuals make informed choices about their coffee consumption habits. Public health initiatives should consider these dual effects to provide comprehensive recommendations that optimize health benefits while mitigating potential drawbacks [18,19,20].

This study also verified the correlation of sleep with markers of FBG, HbA1c, and LDL-C in women. The mechanism by which sleep can affect glycemia and LDL-C involves multiple physiological processes. During deep sleep, cortisol levels decrease, which leads to reduced hepatic glucose production and enhanced insulin sensitivity, allowing for more efficient glucose uptake by muscle and adipose cells [49,50]. This regulation of hormones, including insulin and cortisol, is crucial for maintaining stable blood glucose levels [51,52,53]. Sleep deprivation can lead to insulin resistance, where cells become less responsive to insulin, causing elevated blood glucose levels. Adequate sleep also reduces oxidative stress and chronic inflammation by decreasing the production of pro-inflammatory cytokines and promoting the activity of anti-inflammatory biomarkers [51]. Additionally, sleep influences lipid metabolism by regulating hormones that affect lipid synthesis and breakdown (insulin, leptin, ghrelin, cortisol, adiponectin, and growth hormone). Insufficient sleep can increase the production of lipids and cholesterol by the liver, leading to higher levels of LDL-C [51]. Conversely, poor sleep was linked to lower HDL-C levels [52]. Proper sleep helps balance lipid metabolism, reducing LDL-C levels and increasing HDL-C levels, which is responsible for transporting cholesterol from the arteries to the liver for excretion [32,51,53].

All women in this study were classified as postmenopausal based on a combination of self-reported reproductive histories, the presence of amenorrhea, and age criteria (mean age = 61.8 ± 8.1 years), all of which align with widely accepted clinical thresholds for menopausal status [54]. One of the most impactful shifts is the decline in estrogen levels, which plays a vital role in lipid metabolism, glucose homeostasis, and vascular health [55]. Estrogen helps regulate LDL-C by enhancing hepatic LDL receptor expression, promoting cholesterol clearance, and maintaining a favorable lipid profile. As estrogen levels decrease, LDL-C tends to rise, increasing cardiovascular risk. Additionally, estrogen has anti-inflammatory properties and modulates insulin sensitivity, meaning its decline can exacerbate glycemic dysregulation, possibly explaining the correlations observed between FBG, HbA1c, and sleep duration [55]. However, it is important to emphasize that, in this study, menopausal status was not confirmed via laboratory analysis of circulating sex hormones.

Menopause is further associated with sleep disturbances, including a shorter sleep duration and more frequent nocturnal awakenings, likely driven by disruptions in melatonin production and circadian rhythm instability [56,57]. Estrogen influences neurotransmitter activity involved in sleep regulation, such as serotonin and gamma-aminobutyric acid (GABA), both of which contribute to maintaining sleep quality and stability. Consequently, its decline can lead to fragmented sleep and increased nocturnal arousals. Additionally, evidence suggests that women metabolize caffeine differently than men, potentially due to variations in the activity of the enzyme CYP1A2, which is responsible for caffeine metabolism in the liver. This difference could make women more sensitive to the stimulating effects of coffee, contributing to the correlations observed between coffee consumption, sleep duration, and metabolic markers [56].

A possible explanation for the absence of significant associations in men may be linked to hormonal and metabolic differences that influence physiological responses to coffee consumption. There is evidence that the activity of the CYP1A2 enzyme may be more homogeneous in men, allowing for more stable metabolization of the substance and reducing variability in its effects. Behavioral factors, such as a greater tolerance to coffee’s impact on sleep or differences in the perception of its effects, may have also contributed to the observed results, reinforcing the need for further research into the potential interactions between coffee consumption, metabolism, and biological sex [55,56].

Although coffee is often praised for its potential LDL-C-lowering effects, this study simultaneously revealed elevations in FBG, HbA1c, body weight, and BMI in both sexes—highlighting a paradox within its metabolic profile. Several hypotheses may help reconcile these findings. First, coffee was consumed within a context of ultra-processed, high-calorie dietary patterns, which may have offset its beneficial properties. Second, reverse causation cannot be ruled out: individuals with worsening metabolic indicators may have increased their coffee intake in an attempt to mitigate perceived risks. Additionally, sex-specific hormonal variables—such as estradiol, progesterone, follicle-stimulating hormones, luteinizing hormones, cortisol, and insulin—were not directly measured, but they likely influenced the metabolic responses, especially in postmenopausal women or those with dysregulated hypothalamic–pituitary–adrenal (HPA) axis function. The routine use of statins, such as simvastatin, further complicates interpretation, as LDL-C reductions may have been pharmacologically driven rather than nutritionally mediated. Finally, unmeasured lifestyle factors—including psychological stress, sleep hygiene, and genetic variability—could act as confounding variables. Collectively, these considerations suggest that the observed outcomes may be more reflective of complex behavioral, hormonal, and pharmacological interactions than the isolated effect of coffee consumption itself.

This research has some limitations. Its observational design prevents causal inference, and despite controlling for diet, physical activity, and medications, factors such as genetic predisposition, unpredictable adverse events, and random variation may have influenced the results. Self-reported data on coffee intake and sleep quality are vulnerable to reporting bias, especially given the cohort’s high consumption of ultra-processed foods. The uniform claim of ≤2 g of sugar per serving seems underestimated and likely reflects systematic underreporting. The small sample size and absence of a control group limit generalizability, and the lack of objective monitoring of statin adherence raises the possibility that observed effects may be pharmacological rather than dietary. No stratification by medication type or compliance to it was performed, and sex-specific analyses relied on inferred menopausal status rather than biochemical confirmation.

Future studies should use randomized controlled trials to establish causality and adjust for confounding factors. Broader population samples will increase applicability. It is also crucial to examine the biological mechanisms behind coffee’s impact on metabolic health and sleep—particularly individual variation in caffeine metabolism shaped by genetics, sex, and age. The exclusive use of subjective dietary tools (FFQs and 24-h recalls), without biomarkers like plasma caffeine or paraxanthine, may compromise exposure assessments. An Analysis of coffee with and without sugar should be included. The increase in average coffee consumption from 4.17 to 5.41 cups/day (*p* > 0.05) was not evaluated through longitudinal within-subject analysis, limiting the assessment of dose–response relationships.

Advanced statistical approaches such as mixed-effects models or repeated-measures correlations could better reveal individual trends. Objective sleep monitoring (e.g., actigraphy and portable polysomnography) and an analysis of inflammatory and hormonal biomarkers (TNF-α, IL-6, adiponectin, cortisol, sex hormones) would enhance result precision. Longer follow-up periods would clarify long-term effects, and future studies should explore the timing and preparation methods of coffee intake. Different types of coffee—such as gourmet, specialty, and others—also need to be tested.

Despite its limitations, this study provides valuable insights into the possible multifaceted effects of coffee consumption on metabolic and sleep-related parameters in individuals with T2D and hypertension—especially among postmenopausal women. As the most widely consumed beverage in the world after water, coffee holds significant cultural, social, and nutritional relevance, making it an essential subject of scientific investigation. The strong sex-specific associations observed highlight a potential dual effect of caffeine: a stimulant with sleep-disrupting potential yet containing bioactive compounds that may favor lipid metabolism. Importantly, even when consumed with sugar and within the context of an ultra-processed, hypercaloric diet, traditional coffee demonstrated metabolic benefits, suggesting a protective role that could be possible under certain physiological and lifestyle conditions. However, this possibility depends on individual metabolic responses, hormonal factors, and consumption patterns such as the timing, dosage, and preparation method. The 12-month study design, incorporating quarterly assessments and careful control of lifestyle factors, lends greater credibility and relevance to the findings. By focusing on a socially vulnerable population, the research underscores the importance of tailoring dietary recommendations and public health strategies to specific contextual realities. These insights carry substantial implications for public health, particularly for individuals living with metabolic disorders such as T2D and hypertension. By acknowledging the distinct challenges these populations face, this study underscores the critical need for tailored, context-sensitive dietary strategies and health policies. The findings contribute to advancing sustainable and health-conscious coffee consumption on a global scale.

## 5. Conclusions

A higher coffee intake was significantly associated with a shorter sleep duration in postmenopausal women with T2D and hypertension, reaffirming caffeine’s capacity to disrupt sleep architecture. Simultaneously, coffee consumption demonstrated potential metabolic benefits—particularly reductions in LDL-C levels—even when consumed in traditional, sweetened forms. Moreover, a longer sleep duration correlated with improved glycemic control, as evidenced by lower FBG and HbA1c levels, suggesting a synergistic relationship between sleep quality and metabolic health. These findings underscore the dual effects of coffee—metabolically favorable yet potentially detrimental to sleep—especially among hormonally sensitive populations. Clinically and from a public health perspective, this highlights the necessity of personalized dietary guidance that considers not only the quantity and quality of coffee consumed but also its timing, preparation method, and individual metabolic responsiveness. Given coffee’s cultural ubiquity and complex physiological impact, health guidelines should be nuanced, evidence-based, and context-aware—particularly for individuals with T2D and hypertension. As this study identifies correlations rather than causal relationships, further randomized controlled trials are needed to elucidate the biological mechanisms, evaluate long-term outcomes, and explore sex- and hormone-related variations in metabolic and sleep responses.

## Figures and Tables

**Table 1:** Information about research participants was obtained during the initial study visit.

**Gender**	**Number and Percentage**
Female	20 (50.0%)
Male	20 (50.0%)
**Race**	**Number and Percentage**
White	10 (25%)
Brown	14 (35%)
Black	16 (40%)
**Socioeconomic Status (Class)**	**Number and Percentage**
Class C	26 (65.0%)
Class D	14 (35.0%)
**Age (Years)**	**Mean ± SD**
Male	62.2 ± 8.0
Female	61.8 ± 8.1

Class A (those who earn more than 20 minimum wages); Class B (from 10 to 20 minimum wages); Class C (from 4 to 10 minimum wages); Class D (from 2 to 4 minimum wages); and Class E (receives up to 2 minimum wages). Race classifications were based on self-identification and aligned with the categories. ‘White’ refers to individuals with lighter skin tones of European descent; ‘Black’ refers to individuals with darker skin tones of African descent, and ‘Brown’ includes individuals of mixed racial heritage, commonly a combination of European, African, and Indigenous ancestry. Gaussian data are expressed as the mean ± the standard deviation (SD).

**Table 2:** Parameter data of research participants at the initial visit and the twelfth month (separated by sex).

Parameter	Gender	Baseline	12 Months	*p*-Value
Sedentary (number and percentage)	Women and Men	40 (100%)	40 (100%)	N/A
Fasting blood glucose (mg/dL) (median ± IQR)	Women	159.0 (196.5–132.0)	187.0 (215.9–155.3)	0.0028 *
	Men	160.0 (198.0–135.0)	189.0 (217.0–157.0)	0.0031 *
Glycated hemoglobin (HbA1c) (%) (median ± IQR)	Women	8.7 (9.3–7.3)	9.3 (10.2–7.8)	<0.001 *
	Men	9.8 (11.4–7.4),	10.4 (12.3–7.9)	<0.001 *
Total cholesterol (mg/dL) (median ± IQR)	Women	171.5 (199.8–142.0)	179.5 (195.8–152.5)	0.232
	Men	173.0 (201.0–144.0)	181.0 (197.0–155.0)	0.245
LDL cholesterol (mg/dL) (mean ± SD)	Women	102.4 ± 15.1	100.7 ± 16.8	>0.999
	Men	104.0 ± 16.0	102.0 ± 17.0	>0.999
HDL cholesterol (mg/dL) (median ± IQR)	Women	43.0 (49.5–35.5)	39.0 (43.0–34.0)	0.081
	Men	44.0 (50.0–36.0)	40.0 (44.0–35.0)	0.073
Serum triglycerides (mg/dL) (median ± IQR)	Women	165.5 (201.5–154.0)	175.5 (205.8–140.0)	>0.999
	Men	167.0 (203.0–156.0)	177.0 (207.0–142.0)	>0.999
Body weight (kg) (median ± IQR)	Women	87.7 (98.0–71.5)	92.5 (103.3–73.5)	<0.001 *
	Men	89.0 (100.0–73.0)	94.0 (105.0–75.0)	<0.001 *
BMI (kg/m2) (median ± IQR)	Women	32.0 (35.5–30.2)	34.0 (37.0–30.0)	<0.001 *
	Men	33.0 (36.0–30.0)	35.0 (38.0–30.1)	<0.001 *
Waist circumference (cm) (mean ± SD)	Women	106.8 ± 12.0	110.6 ± 13.1	0.090
	Men	108.0 ± 13.0	112.0 ± 14.0	0.060
Waist-to-hip ratio (unitless) (median ± IQR)	Women	1.0 (1.1–1.0)	1.0 (1.2–1.0)	0.030 *
	Men	1.1 (1.2–1.0)	1.2 (1.3–1.0)	0.050 *
Systolic blood pressure (mmHg) (mean ± SD)	Women	145.3 ± 11.0	147.6 ± 16.4	0.990
	Men	146.0 ± 12.0	148.0 ± 17.0	0.970
Diastolic blood pressure (mmHg) (median ± IQR)	Women	88.5 (98.0–87.0)	89.5 (95.7–86.1)	>0.999
	Men	89.0 (99.0–88.0)	90.0 (96.0–87.0)	>0.970
Heart rate (bpm) (median ± IQR)	Women	87.0 (89.9–78.9)	87.2 (91.7–78.6)	>0.999
	Men	88.0 (90.0–79.0)	88.2 (92.0–79.5)	>0.999
BMR (kcal) (median ± IQR)	Women	1206 (1408–1204)	1226 (1428–1224)	>0.999
	Men	1410 (1512–1208)	1440 (1542–1248)	>0.999
TEE (kcal) (median ± IQR)	Women	1565 (1689–1446)	1575 (1679–1476)	>0.999
	Men	1770 (1894–1450)	1780 (1884–1480)	>0.999
TEV (kcal) (median ± IQR)	Women	1910 (2103–1904)	1930 (2173–1974)	>0.999
	Men	2120 (2310–1910)	2140 (2370–1970)	>0.999
Sleep duration (hours/night)	Women	7.0 ± 2.133	6.5 ± 3.191	<0.005 *
	Men	7.1 ± 2.040	6.4 ± 3.01	<0.005 *
Nocturnal awakenings (unit per night)	Women	1.0 ± 0.5	2.0 ± 0.6	<0.001 *
	Men	1.2 ± 0.3	2.5 ± 0.2	<0.001 *
Difficulty falling asleep (percentage and number)	Women	75% (15)	80% (16)	N/A
	Men	85% (17)	90% (18)	N/A

Basal metabolic rate (BMR); total energy expenditure (TEE); total energetic value (TEV). Non-normal data are expressed as the median ± the interquartile range (IQR); normal data are expressed as the mean ± the standard deviation (SD). An asterisk (*) indicates a significant difference between values observed at the first visit and the twelfth month (the Friedman test followed by the Durbin–Conover post hoc test for non-parametric data; a repeated measures ANOVA followed by Tukey’s post hoc test for parametric data; * *p* < 0.05). N/A = not applicable. No significant differences were observed between sexes.

**Table 3:** Coffee consumption and the type of coffee between baseline and the 12th month.

Parameter	Gender	Baseline	12 Months	*p*-Value
Coffee consumption (number and percentage)	Women	20 (100%)	20 (100%)	N/A
	Men	20 (100%)	20 (100%)	N/A
		**Mean ± SD** **Baseline**	**Mean ± SD** **12 Months**	
Cups of coffee (unit)	Women	4.29 ± 0.317	5.81 ± 0.298	>0.05
	Men	4.05 ± 0.398	5.00 ± 0.333	>0.05
		**Baseline**	**12 Months**	
Type of coffee consumed (traditional, specialty, or gourmet) (number and percentage)	Women	Traditional 20 (100%)	Traditional 20 (100%)	N/A
	Men	Traditional 20 (100%)	Traditional 20 (100%)	N/A
		**Baseline**	**12 Months**	N/A
Sweetened? (number and percentage)	Women	Sugar 20 (100%)	Sugar 20 (100%)	N/A
	Men	Sugar 20 (100%)	Sugar 20 (100%)	N/A
		**Baseline**	**12 Months**	N/A
How much sugar? (number and percentage)	Women	≤2 Teaspoons per cup (≤2 g) 20 (100%)	≤2 Teaspoons per cup (≤2 g) 20 (100%)	N/A
Coffee intake time? Before 15 h or after 16 h (number and percentage)	Women	Before 15 h 20 (100%)	Before 15 h 20 (100%)	N/A
	Men	Before 15 h 20 (100%)	Before 15 h 20 (100%)	N/A

N/A = not applicable. Note: “Traditional coffee” typically refers to the everyday coffee consumed in households and cafes, using basic brewing methods such as drip coffee makers, French press, or espresso machines, often pre-ground or instant coffee. “Specialty coffee” is high-quality coffee sourced and roasted carefully, often from single-origin sources, and brewed using methods that highlight unique flavors, like pour-over or AeroPress. “Gourmet coffee” refers to premium blends, flavored coffees, or beverages made with high-quality ingredients, often offering unique flavors or ingredients not found in regular coffee. Note: For cups of coffee, each cup contains 50 mL; and 1 teaspoon = 1 g.

**Table 4:** Coffee brands, preparation method, and preferences among participants over 12 months.

Coffee Brand	Total Participants	Female	Male	Preparation Method (Tablespoons Per Liter)	Caffeine Content (%)
3 Corações^®^	15	8	7	6	1.4 to 1.6
Pilão^®^	12	6	6	5	1.4 to 1.8
Melitta^®^	10	5	5	6	1.4 to 1.7
Others (Cimo^®^, Caboclo^®^, and Pelé^®^)	3	1	2	7	1.3 to 1.6
Total	40	20	20		

Pilão^®^ (Natal, Rio Grande do Norte, Brazil); Melitta^®^ (Varginha, Minas Gerais, Brazil); 3 Corações^®^ (Eusébio, Ceará, Brazil); Cimo^®^ (São José do Rio Preto, São Paulo, Brazil); Caboclo^®^ (Salvador, Bahia, Brazil); and Pelé^®^ (Salvador, Bahia, Brazil). Note: Caffeine Content: This refers to the amount of caffeine present in the coffee, expressed as a percentage of the total weight of the ground coffee. For example, 100 g of ground coffee contains 1.4% caffeine, which means that 1.4 g of the total weight is composed of caffeine. Variations: The variation from 1.3% to 1.8% can be due to different coffee batches, cultivation methods, and roasting and preparation techniques. Concentration: For most people, a caffeine concentration between 1.3% and 1.8% is considered moderate. A 50 mL cup of coffee with ground coffee containing 1.4% caffeine would have approximately 140 mg of caffeine [4,5,6].

## Data Availability

The data were gathered and managed using the REDCap 14.0.9 electronic data capture tools, which are hosted at REDCap—FUNFARME/FAMERP (from the State Faculty of Medicine). All data can be made available upon request to the corresponding author. However, due to privacy considerations, the data are not available for public access.

## Supplementary Materials

The following supporting information can be downloaded at: https://www.mdpi.com/article/10.3390/biomedicines13081875/s1, Table S1: Diet assessment data of research participants, obtained during the study; Table S2: Diet assessment data of research participants, obtained during the study—Separated by sex; Table S3: Health parameters data of research participants obtained during the study (baseline vs. 12th visit); Table S4: Linear Regression, Pearson correlation, and Spearman analysis (health parameters vs. sleep and coffee) obtained during baseline study-Women; Table S5: Linear regression, Pearson correlation, and Spearman analysis (health parameters vs. sleep and coffee) obtained during baseline study—Man; Table S6: Linear regression, Pearson correlation, and Spearman (health parameters vs. sleep and coffee) obtained during 12th month study—Women; Table S7: Linear regression, Pearson correlation, and Spearman (health parameters vs. sleep and coffee) obtained during 12th month study-Man; Figure S1: Fasting glycemia (mg/dL) and Glycated hemoglobin (%) over 12 months; Figure S2: Total cholesterol (mg/dL), LDL cholesterol (mg/dL), HDL cholesterol (mg/dL), and Triglycerides (mg/dL) over 12 months; Figure S3: Weight (kg), Body mass index (BMI) (kg/m^2^), Waist circumference (cm), and Waist-to-hip ratio (WHR) (unit) over 12 months; Figure S4: Blood pressure (mmHg) and Heart rate (bpm) over 12 months; Figure S5: Night awakens (unit) and Sleep duration (h) over 12 months.

## Abbreviations

The following abbreviations are used in this manuscript:

**Acronym**	**Meaning**
**BMI**	Body Mass Index
**BP**	Blood Pressure
**BMR**	Basal Metabolic Rate
**CAAE**	Certificate of Presentation for Ethical Review
**CAPES**	Coordination for the Improvement of Higher Education Personnel
**CNPq**	National Council for Scientific and Technological Development
**CYP1A2**	Cytochrome P450 1A2
**CYP7A**	Cholesterol 7-alpha-hydroxylase
**DV**	Dependent Variable
**FBG**	Fasting Blood Glucose
**FFQ**	Food Frequency Questionnaire
**GABA**	Gamma-Aminobutyric Acid
**GLP-1**	Glucagon-Like Peptide-1
**HbA1c**	Glycated Hemoglobin
**HDL-C**	High-Density Lipoprotein Cholesterol
**HMG-CoA**	3-Hydroxy-3-Methylglutaryl-Coenzyme A
**HPA**	Hypothalamic–pituitary–adrenal
**IQR**	Interquartile Range
**LDL-C**	Low-Density Lipoprotein Cholesterol
**NCT**	Clinical Trial Registry Number
**PPARα**	Peroxisome Proliferator-Activated Receptor Alpha
**PSQI**	Pittsburgh Sleep Quality Index
**R^2^**	Coefficient of Determination
**REDCap**	Research Electronic Data Capture
**SD**	Standard Deviation
**SGLT-2**	Sodium-Glucose Transport Protein 2
**SUS**	Unified Health System
**SREBP-2**	Sterol Regulatory Element-Binding Protein 2
**T2D**	Type 2 Diabetes
**TEE**	Total Energy Expenditure
**TEV**	Total Energetic Value
**TG**	Triglycerides
**TNF-α**	Tumor Necrosis Factor Alpha
**WC**	Waist Circumference
**WHR**	Waist-to-Hip Ratio

## References

[1] Muñoz-Pajares A.J., Várzea V., Silva M.D.C. (2023). The story of coffee: Legend and truth. Trends Plant Sci..

[2] Jamil S., Raza M.L., Naqvi S., Zehra A. (2024). Behavioral and psychological aspects of coffee consumption. Prog. Brain Res..

[3] Simões M.B.A., Brandão J.M., Antunes A.B.S., Sichieri R. (2024). Coffee intake in Brazil influences the consumption of sugar, sweets, and beverages. Nutrients.

[4] Ferreira J.C., Gomes M.S., Oliveira M.L., Santos L.D. (2023). Coffee fermentation process: A review. Food Res. Int..

[5] Santos V.P., Ribeiro P.C.C., Rodrigues L.B. (2023). Sustainability assessment of coffee production in Brazil. Environ. Sci. Pollut. Res. Int..

[6] Volsi B., Telles T.S., Caldarelli C.E., Camara M.R.G.D. (2019). The dynamics of coffee production in Brazil. PLoS ONE.

[7] Rai S.P., Ansari A.H., Singh D., Singh S. (2024). Coffee, antioxidants, and brain inflammation. Prog. Brain Res..

[8] Surma S., Romańczyk M., Filipiak K.J., Lip G.Y.H. (2023). Coffee and cardiac arrhythmias: Update review of the literature and clinical studies. Cardiol. J..

[9] Raeis-Abdollahi E., Raise-Abdullahi P., Rashidy-Pour A., Meamar M., Askari H. (2024). Coffee’s protective mechanisms against neurodegeneration. Prog. Brain Res..

[10] Manghi P., Bhosle A., Wang K., Marconi R., Selma-Royo M., Ricci L., Asnicar F., Golzato D., Ma W., Hang D. (2024). Coffee consumption is associated with intestinal *Lawsonibacter asaccharolyticus* abundance and prevalence across multiple cohorts. Nat. Microbiol..

[11] Baspinar B., Eskici G., Ozcelik A.O. (2017). How coffee affects metabolic syndrome and its components. Food Funct..

[12] Iriondo-DeHond A., Iriondo-DeHond M., del Castillo M.D. (2020). Applications of Compounds from Coffee Processing By-Products. Biomolecules.

[13] Tahmouzi S., Nasab S.S., Alizadeh-Salmani B., Zare L., Mollakhalili-Meybodi N., Nematollahi A. (2024). Coffee substitutes: A review of the technology, characteristics, application, and future perspective. Compr. Rev. Food Sci. Food Saf..

[14] Yeager S.E., Batali M.E., Guinard J.X., Ristenpart W.D. (2023). Acids in coffee: A review of sensory measurements and meta-analysis of chemical composition. Crit. Rev. Food Sci. Nutr..

[15] Costa G.X.R., Silva L.C.F., Oliveira L.M., Santos L.D. (2024). Microbiota of Arabica coffee: Insights from soil to fruit. World, J. Microbiol. Biotechnol..

[16] Clark I., Landolt H.P. (2017). Coffee, caffeine, and sleep: A systematic review of epidemiological studies and randomized controlled trials. Sleep Med. Rev..

[17] Gardiner C., Weakley J., Burke L.M., Roach G.D., Sargent C., Maniar N., Townshend A., Halson S.L. (2023). The effect of caffeine on subsequent sleep: A systematic review and meta-analysis. Sleep Med. Rev..

[18] Bagheri Davisaraei Y., Nateghi S., Rashidipour H., Raise-Abdullahi P., Rashidy-Pour A. (2024). Coffee and sleep: Benefits and risks. Prog. Brain Res..

[19] Kim E.J., Hoffmann T.J., Nah G., Vittinghoff E., Delling F., Marcus G.M. (2021). Coffee consumption and incident tachyarrhythmias: Reported behavior, Mendelian randomization, and their interactions. JAMA Intern. Med..

[20] Yang Y., Wu J., Li S., Yu W., Zhu H., Wang Y., Li Y. (2023). Smoking, Coffee Consumption, Alcohol Intake, and Obstructive Sleep Apnea: A Mendelian Randomization Study. Curr. Neurovascular Res..

[21] van der Linden M., Olthof M.R., Wijnhoven H.A.H. (2023). The association between caffeine consumption from coffee and tea and sleep health in male and female older adults: A cross-sectional study. Nutrients.

[22] Cornelis M.C., O’Donnell C.J. (2019). The impact of caffeine and coffee on human health. Nutrients.

[23] Grosso G., Azpeitia G.G., Súarez D.R., Rodríguez A.S., Ferrer J.F.L., Serra-Majem L. (2017). Factors Associated with Stunting among Children Aged 0 to 59 Months from the Central Region of Mozambique. Nutrients.

[24] Chen Y., Zhang Y., Zhang M., Yang H., Wang Y. (2022). Consumption of coffee and tea with all-cause and cause-specific mortality: A prospective cohort study. BMC Med..

[25] Nehlig A. (2022). Effects of coffee on the gastro-intestinal tract: A narrative review and literature update. Nutrients.

[26] Miranda A.M., Steluti J., Goulart A.C., Benseñor I.M., Lotufo P.A., Marchioni D.M. (2018). Coffee consumption and coronary artery calcium score: Cross-sectional results of ELSA-Brasil (Brazilian Longitudinal Study of Adult Health). J. Am. Heart Assoc..

[27] Moran-Lev H., Cohen S., Zelber-Sagi S., Mayer E.M., Anafy A., Yerushalmy-Feler A., Lubetzky R. (2023). Effect of Coffee and Tea Consumption on Adolescent Weight Control: An Interventional Pilot Study. Child. Obes..

[28] Miranda A.M., Goulart A.C., Benseñor I.M., Lotufo P.A., Marchioni D.M. (2021). Coffee consumption and risk of hypertension: A prospective analysis in the cohort study. Clin. Nutr..

[29] Minari T.P., Manzano C.F., Yugar L.B.T., Sedenho-Prado L.G., Rubio T.d.A., Tácito L.H.B., Pires A.C., Vilela-Martin J.F., Cosenso-Martin L.N., Ludovico N.D. (2024). Demystifying Obesity: Understanding, Prevention, Treatment, and Stigmas. Nutr. Rev..

[30] Minari T.P., Tácito L.H.B., Yugar L.B.T., Ferreira-Melo S.E., Manzano C.F., Pires A.C., Moreno H., Vilela-Martin J.F., Cosenso-Martin L.N., Yugar-Toledo J.C. (2023). Nutritional Strategies for the Management of Type 2 Diabetes Mellitus: A Narrative Review. Nutrients.

[31] Faul F., Erdfelder E., Lang A.G., Buchner A. (2007). Power 3.1: A flexible statistical power analysis program for the social, behavioral, and biomedical sciences. Behav. Res. Methods.

[32] American Diabetes Association Professional Practice Committee (2025). 10. Cardiovascular disease and risk management: Standards of care in diabetes—2025. Diabetes Care.

[33] Feitosa A.D.M., Barroso W.K.S., Junior D.M., Nobre F., Mota-Gomes M.A., Jardim P.C.B.V., Amodeo C., Oliveira A.C., Alessi A., Sousa A.L.L. (2024). Diretrizes Brasileiras de Medidas da Pressão Arterial Dentro e Fora do Consultório—2023. Arq. Bras. Cardiol..

[34] Bertolazi A.N., Fagondes S.C., Hoff L.S., Dartora E.G., Miozzo I.C.d.S., de Barba M.E.F., Barreto S.S.M. (2011). Validation of the Brazilian Portuguese version of the Pittsburgh Sleep Quality Index. Sleep Med..

[35] Lima L.S., Araujo M.A., Ornelas G.C., Logrado M.H. (2012). Validação de instrumento de triagem nutricional [Validation of a nutritional screening tool]. Acta Med. Port..

[36] Ferraroni M., Tavani A., Decarli A., Franceschi S., Parpinel M., Negri E., La Vecchia C. (2004). Reproducibility and validity of coffee and tea consumption in Italy. Eur. J. Clin. Nutr..

[37] Harris P.A., Taylor R., Minor B.L., Elliott V., Fernandez M., O’Neal L., McLeod L., Delacqua G., Delacqua F., Kirby J. (2019). The REDCap consortium: Building an international community of software platform partners. J. Biomed. Inform..

[38] Kasza J., Wolfe R. (2014). Interpretation of commonly used statistical regression models. Respirology.

[39] Schober P., Boer C., Schwarte L.A. (2018). Correlation coefficients: Appropriate use and interpretation. Anesth. Analg..

[40] The Jamovi Project (2023). Jamovi (Version 2.6.23) [Computer Software]. https://www.jamovi.org.

[41] Wang X., Ma H., Sun Q., Li J., Heianza Y., Van Dam R.M., Hu F.B., Rimm E., E Manson J., Qi L. (2025). Coffee drinking timing and mortality in US adults. Eur. Hear. J..

[42] Marcus G.M., Rosenthal D.G., Nah G., Vittinghoff E., Fang C., Ogomori K., Joyce S., Yilmaz D., Yang V., Kessedjian T. (2023). Acute Effects of Coffee Consumption on Health among Ambulatory Adults. N. Engl. J. Med..

[43] Gu X., Zhang S., Ma W., Wang Q., Li Y., Xia C., Xu Y., Zhang T., Yang L., Zhou M. (2022). The Impact of Instant Coffee and Decaffeinated Coffee on the Gut Microbiota and Depression-Like Behaviors of Sleep-Deprived Rats. Front. Microbiol..

[44] Pereira G.V.M., de Carvalho Neto D.P., Magalhães Júnior A.I., do Prado F.G., Pagnoncelli M.G.B., Karp S.G., Soccol C.R. (2020). Chemical composition and health properties of coffee and coffee by-products. Adv. Food Nutr. Res..

[45] Park J., Han J.W., Lee J.R., Byun S., Suh S.W., Kim T., Yoon I.Y., Kim K.W. (2018). Lifetime coffee consumption, pineal gland volume, and sleep quality in late life. Sleep.

[46] Barrea L., Pugliese G., Frias-Toral E., El Ghoch M., Castellucci B., Chapela S.P., Carignano M.d.L.A., Laudisio D., Savastano S., Colao A. (2021). Coffee consumption, health benefits and side effects: A narrative review and update for dietitians and nutritionists. Crit. Rev. Food Sci. Nutr..

[47] van Dam R.M., Hu F.B., Willett W.C. (2020). Coffee, caffeine, and health. N. Engl. J. Med..

[48] Surma S., Oparil S. (2021). Coffee and arterial hypertension. Curr. Hypertens. Rep..

[49] Haghighatdoost F., Hajihashemi P., Romeiro A.M.d.S., Mohammadifard N., Sarrafzadegan N., de Oliveira C., Silveira E.A. (2023). Coffee Consumption and Risk of Hypertension in Adults: Systematic Review and Meta-Analysis. Nutrients.

[50] Miller M.A., Howarth N.E. (2023). Sleep and cardiovascular disease. Emerg. Top. Life Sci..

[51] Hoek A.G., van Oort S., Elders P.J.M., Beulens J.W.J. (2022). Causal association of cardiovascular risk factors and lifestyle behaviors with peripheral artery disease: A Mendelian randomization approach. J. Am. Heart Assoc..

[52] Minari T.P., Manzano C.F., Yugar L.B.T., Sedenho-Prado L.G., Rubio T.d.A., Tácito L.H.B., Pires A.C., Vilela-Martin J.F., Cosenso-Martin L.N., Ludovico N.D. (2024). The effect of breakfast skipping and sleep disorders on glycemic control, cardiovascular risk, and weight loss in type 2 diabetes. Clin. Nutr. ESPEN.

[53] Balcioglu S.S.K., Balcioglu Y.H., Balaban O.D. (2022). The association between chronotype and sleep quality, and cardiometabolic markers in patients with schizophrenia. Chronobiol. Int..

[54] Nair A.R., Pillai A.J., Nair N. (2021). Cardiovascular changes in menopause. Curr. Cardiol. Rev..

[55] Gersh F., O’Keefe J.H., Elagizi A., Lavie C.J., Laukkanen J.A. (2024). Estrogen and cardiovascular disease. Prog. Cardiovasc. Dis..

[56] Maki P.M., Panay N., Simon J.A. (2024). Sleep disturbance associated with the menopause. Menopause.

[57] Hachul H., Hachul de Campos B., Lucena L., Tufik S. (2023). Sleep during menopause. Sleep Med. Clin..

