# Supplementary material for: Correlations Between Coffee Intake, Glycemic Control, Cardiovascular Risk, and Sleep in Type 2 Diabetes and Hypertension: A 12-Month Observational Study
Source: Biomedicines. 2025 Aug 1;13(8):1875. doi: 10.3390/biomedicines13081875 (PMC12383952; doi:10.3390/biomedicines13081875)
Supplement: Supplementary file 1 [file biomedicines-13-01875-s001.zip › supplementary materials.pdf]

Supplementary Materials - Tables

**Table S1. Diet assessment data of research participants, obtained during the study.**	**BASELINE**	**12th VISIT**	***p***
Carbohydrate (g) (median ± IQR)	249 (260-249)	255 (265-249)	0.030
Carbohydrate (%) (median ± IQR)	47.1 (51-47)	48.2 (52-48)	< .001
Proteins (g) (median ± IQR)	63.2 (63.2-60.2)	62 (67-61)	0.657
Proteins (%) (median ± IQR)	16.2 (16.8-15.6)	15.8 (16.5-15.2)	0.805
Total Fats (g) (median ± IQR)	102 (102-100)	129 (154-111)	< .001
Total Fats (%) (median ± IQR)	35.9 (37.7-34.1)	36.3(37.6-35)	0.992
Saturated Fats (g) (median ± IQR)	48.1 (48.1-36.4)	48.2 (48.4-48.1)	< .001
Monounsaturated Fats (g) (median ± IQR)	14.6 (19.6-14.2)	13.1 (13.4-13.1)	< .001
Polyunsaturated Fats (g) (median ± IQR)	2.7 (8.4-2.5)	1.9 (2.5-1.9)	< .001
Trans Unsaturated Fats (g) (median ± IQR)	2.5 (2.9-2.5)	2.5 (2.5-2.4)	0.326
Fiber (g) (mean ± SD)	7.2 ±7	7.3 ±7.2	0.442
Omega 3 (g) (median ± IQR)	0	0	N/A
Omega 6 (g) (median ± IQR)	0.950 (1.1-0.9)	1 (1.1-1)	0.997
Cholesterol (mg) (median ± IQR)	140 (172-122)	203 (203-189)	0.009
Sodium (mg) (median ± IQR)	4562 (5645-4301)	4342 (4565-4301)	< .001
Potassium (mg) (median ± IQR)	614 (2012-363)	523 (576-501)	< .001
Calcium (mg) (median ± IQR)	451 (472-427)	467 (472-454)	0.999
Magnesium (mg) (median ± IQR)	161(177-120)	101 (120-101)	0.006
Iron (mg) (median ± IQR)	15 (25.9-11.8)	24 (25.9-23)	< .001
Zinc (mg) (median ± IQR)	5 (6.4-2.72)	2.72 (2.80-2.60)	< .001
Vitamin D (mcg) (median ± IQR)	0.7(0.8-0.7)	0.5 (0.575-0.3)	< .001
Vitamin K (mcg) (median ± IQR)	0.2 (0.2-0.225)	0.2 (0.2-0.1)	0.998
Vitamin C (mcg) (median ± IQR)	0	0	N/A
Vitamin A (mcg) (median ± IQR)	500 (500-500)	476 (500-476)	0.035
Cyanocobalamin Vitamin b12 (mcg) (median ± IQR)	3 (3-2)	0.9 (1.01-0.9)	< .001

Non-parametric data expressed as median ± interquartile range (IQR); Parametric data expressed as mean ± standard deviation (SD). Friedman test followed by Durbin-Conover post hoc test for non-parametric data; Repeated measures ANOVA followed by Tukey post hoc test for parametric data; *$p < 0.05$. N/A= Not Applicable.

**Table S2. Diet assessment data of research participants, obtained during the study – Separated by sex**	**Sex**	**Baseline (Median ± IQR)**	**12th Visit (Median ± IQR)**	***p*-Value**
Carbohydrate (g) (median ± IQR)	Male	270 (280-260)	275 (285-260)	0.028
	Female	230 (240-225)	235 (245-225)	0.032
Carbohydrate (%) (median ± IQR)	Male	48.5 (52-48)	49.3 (53-48.5)	< .001
	Female	45.8 (50-45)	46.7 (51-46)	< .001
Proteins (g) (median ± IQR)	Male	70 (75-68)	68 (72-66)	0.612
	Female	55 (58-53)	54 (57-52)	0.659
Proteins (%) (median ± IQR)	Male	17.5 (18-16.5)	16.9 (17.5-16)	0.798
	Female	15.4 (16-14.8)	15.1 (15.8-14.5)	0.804
Total Fats (g) (median ± IQR)	Male	110 (115-105)	135 (160-120)	< .001
	Female	95 (100-90)	120 (140-105)	< .001
Total Fats (%) (median ± IQR)	Male	36.5 (38-35)	37 (38-35.5)	0.995
	Female	35.4 (37-34)	35.9 (37-35)	0.990
Saturated Fats (g) (median ± IQR)	Male	50 (50-38)	51 (51-50)	< .001
	Female	46 (47-35)	47 (48-46)	< .001
Monounsaturated Fats (g) (median ± IQR)	Male	16 (20-15)	14 (14-13)	< .001
	Female	13 (18-12)	12 (13-12)	< .001
Polyunsaturated Fats (g) (median ± IQR)	Male	3 (9-3)	2.2 (2.8-2.1)	< .001
	Female	2.5 (8-2.3)	1.7 (2.3-1.7)	< .001
Trans Unsaturated Fats (g) (median ± IQR)	Male	2.6 (3-2.6)	2.5 (2.6-2.5)	0.326
	Female	2.4 (2.8-2.4)	2.4 (2.5-2.3)	0.326
Fiber (g) (Mean ± SD)	Male	7.5 ± 7.2	7.6 ± 7.3	0.442
	Female	7.0 ± 6.8	7.1 ± 7.0	0.442
Omega 3 (g)	Male	0	0	N/A
	Female	0	0	N/A
Omega 6 (g) (median ± IQR)	Male	1.0 (1.2-1.0)	1.1 (1.2-1.1)	0.997
	Female	0.9 (1.1-0.9)	1.0 (1.1-1.0)	0.997

Cholesterol (mg) (median ± IQR)	Male	160 (180-140)	220 (225-200)	0.007
	Female	125 (140-110)	180 (190-170)	0.010
Sodium (mg) (median ± IQR)	Male	4700 (5800-4400)	4500 (4700-4400)	< .001
	Female	4400 (5500-4200)	4300 (4500-4200)	< .001
Potassium (mg) (median ± IQR)	Male	650 (2100-380)	550 (590-530)	< .001
	Female	580 (2000-350)	500 (560-490)	< .001
Calcium (mg) (median ± IQR)	Male	460 (480-440)	480 (490-460)	0.999
	Female	450 (470-430)	460 (470-450)	0.999
Magnesium (mg) (median ± IQR)	Male	170 (180-130)	110 (130-110)	0.006
	Female	160 (175-120)	100 (120-100)	0.006
Iron (mg) (median ± IQR)	Male	18 (27-12)	26 (27-24)	< .001
	Female	14 (25-11)	23 (26-22)	< .001
Zinc (mg) (median ± IQR)	Male	5.5 (6.5-3)	3.0 (3.2-2.8)	< .001
	Female	4.8 (6.2-2.5)	2.7 (2.9-2.6)	< .001
Vitamin D (mcg) (median ± IQR)	Male	0.8 (0.9-0.7)	0.6 (0.7-0.4)	< .001
	Female	0.7 (0.8-0.7)	0.5 (0.6-0.3)	< .001
Vitamin K (mcg) (median ± IQR)	Male	0.2 (0.3-0.2)	0.2 (0.2-0.1)	0.998
	Female	0.2 (0.2-0.22)	0.2 (0.2-0.1)	0.998
Vitamin C (mcg) (median ± IQR)	Male	0	0	N/A
	Female	0	0	N/A
Vitamin A (mcg) (median ± IQR)	Male	510 (520-500)	490 (500-480)	0.035
	Female	500 (500-500)	476 (500-476)	0.035
Vitamin B12 (Cyanocobalamin) (mcg) (median ± IQR)	Male	3.2 (3.5-2.5)	1.2 (1.3-1.0)	< .001
	Female	3 (3-2)	0.9 (1.01-0.9)	< .001

Non-parametric data expressed as median ± interquartile range (IQR); Parametric data expressed as mean ± standard deviation (SD). Friedman test followed by Durbin-Conover post hoc test for non-parametric data; Repeated measures ANOVA followed by Tukey post hoc test for parametric data; *p < 0.05. N/A= Not Applicable.

**Table S3. Health parameters data of research participants obtained during the study (baseline vs. 12th visit)**	**Baseline**	**12th Visit**	***p-Value***
Weight (median ± IQR)	87.7 (98.0–71.5)	92.5 (103.3–73.5) *	<0.05*
BMI (kg/m2) (median ± IQR)	30.0 (35.5–27.2)	32.0 (37.0–28.0) *	<0.05*
WC (cm) (mean ± SD)	106.8 ± 12.0	110.6 ± 13.1	>0.05
FBG (mg/dL) (median ± IQR)	159.0 (196.5–132.0)	187.0 (215.9–155.3) *	<0.05*
HbA1c (%) (median ± IQR)	8.7 (9.3–7.3)	9.3 (10.2–7.8) *	<0.05*
TC (mg/dL) (median ± IQR)	171.5 (199.8–142.0)	179.5 (195.8–152.5)	>0.05
LDL-C (mg/dL) (mean ± SD)	102.4 ± 15.1	100.7 ± 16.8	>0.05
HDL-C (mg/dL) (median ± IQR)	43.0 (49.5–35.5)	39.0 (43.0–34.0)	>0.05
TG (mg/dL) (median ± IQR)	165.5 (201.5–154.0)	175.5 (205.8–140.0)	>0.05
SBP (mmHg) (median ± IQR)	145.3 ± 11.0	147.6 ± 16.4	>0.05
DBP (mmHg) (median ± IQR)	88.5 (98.0–87.0)	89.5 (95.7–86.1)	>0.05
HR (bpm) (median ± IQR)	87.0 (89.9–78.9)	87.2 (91.7–78.6)	>0.05
Sleep duration (h) (mean ± SD)	7.5 ± 0.5	6.45 ± 0.5	<0.05*
Sleep awakens (unit) (mean ± SD)	1.1 ± 0.1	2.25 ± 0.25	<0.05*
Difficulty Falling Asleep (percentage and number)	82.5% (33)	82.5% (33)	N/A
Cup of coffee (unit) (mean ± SD)	4.17±0.360	5.41±0.316	>0.05
BMR (kcal) (median ± IQR)	1306 (1408–1204)	1306 (1408–1204)	>0.9
TEE (kcal) (median ± IQR)	1565 (1689–1446)	1565 (1689–1446)	>0.9
TEV (kcal) (median ± IQR)	1910 (2103–1904)	1910 (2103–1904)	>0.9
Sedentary (percentage and number)	100% (40)	100% (40)	N/A

Body mass index (BMI); Waist circumference (WC); Fasting blood glucose (FBG); Glycated hemoglobin (HbA1c); Total cholesterol (TC); LDL cholesterol (LDL-C); HDL cholesterol (HDL-C); Serum triglycerides (TG); Systolic blood pressure (SBP); Diastolic blood pressure (DBP); Heart Rate (HR); Basal metabolic rate (BMR); Total energy expenditure (TEE); Total energetic value (TEV); Non-parametric data expressed as median ± interquartile range (IQR); Parametric data expressed as mean ± standard deviation (SD). Friedman test followed by Durbin-Conover post hoc test for non-parametric data; Repeated measures ANOVA followed by Tukey post hoc test for parametric data; *$p < 0.05$. N/A= Not Applicable.

**Table S4. Linear Regression, Pearson Correlation, and Spearman analysis (health parameters vs. sleep and coffee) obtained during baseline study - Women**	**Sleep duration**			**Sleep awakens**			**Cup of coffee**			
	Linear Regression (R^2)	Pearson (r)	Spearman (ϱ)	Linear Regression (R^2)	Pearson (r)	Spearman (ϱ)	Linear Regression (R^2)	Pearson (r)	Spearman (ϱ)	***p-Value***
Fasting blood glucose (mg/dL) (median ± IQR)	0.707*	-0.841 (p=0.031)*	-0.832*	0.227	0.089	0.163	0.158	0.154	0.135	>0.05
Glycated hemoglobin (%) (median ± IQR)	0.680*	-0.831 (p=0.037)*	-0.940*	0.266	-0.051	-0.045	0.108	0.102	-0.038	>0.05
Total cholesterol (mg/dL) (median ± IQR)	0.089	-0.050	0.102	0.189	0.067	0.118	0.101	-0.091	0.115	>0.05
LDL cholesterol (mg/dL) (mean ± SD)	0.508*	-0.713 (p=0.050)*	-0.773*	0.151	-0.089	0.068	0.672*	-0.820 (p=0.044)*	-0.885*	>0.05
HDL cholesterol (mg/dL) (median ± IQR)	0.054	-0.077	0.089	0.317	0.039	0.094	0.073	-0.051	0.095	>0.05
Serum triglycerides (mg/dL) (median ± IQR)	0.142	0.091	-0.050	0.357	-0.044	-0.055	0.138	0.092	-0.060	>0.05
Body weight (kg) (median ± IQR)	0.099	-0.013	0.199	0.277	0.058	0.204	0.112	-0.017	0.187	>0.05
BMI (kg/m^2) (median ± IQR)	0.165	0.057	0.098	0.198	-0.078	0.105	0.175	0.071	0.110	>0.05
Waist circumference (cm) (mean ± SD)	0.072	-0.030	0.054	0.282	0.112	0.059	0.085	-0.031	0.065	>0.05
Systolic blood pressure (mmHg) (median ± IQR)	0.128	0.121	-0.077	0.160	-0.062	-0.080	0.144	0.114	-0.070	>0.05
Diastolic blood pressure (mmHg) (median ± IQR)	0.154	-0.040	0.142	0.227	0.089	0.150	0.158	-0.055	0.145	>0.05
Heart rate (bpm) (median ± IQR)	0.102	0.073	0.091	0.266	-0.051	0.098	0.108	0.076	0.105	>0.05
Sleep duration (h)	N/A	N/A	N/A	0.317	0.039	0.095	0.073	-0.051	0.094	>0.05

Coefficient of Determination, (R^2), ranges from 0 to 1. When (R^2) = 1, the model explains 100% of the variation in the data. Conversely, when (R^2) = 0, the model does not explain any of the variation in the data. Pearson Correlation (r): A value indicating the strength and direction of the association between two variables. The value of r represents the correlation coefficient, which ranges between -1 and 1. A value of $r = 1$ indicates a perfect positive correlation, $r = -1$ indicates a perfect negative correlation, and $r = 0$ indicates no correlation. Spearman correlation coefficient, (ρ), measures the strength and direction of association between two ranked variables. It ranges from -1 to 1, where 1 indicates a perfect positive correlation, -1 indicates a perfect negative correlation, and 0 indicates no correlation.

**Table S5. Linear Regression, Pearson Correlation, and Spearman analysis (health parameters vs. sleep and coffee) obtained during baseline study - Man**	**Sleep duration**			**Sleep awakens**			**Cup of coffee**			
	Linear Regression (R^2)	Pearson (r)	Spearman (ρ)	Linear Regression (R^2)	Pearson (r)	Spearman (ρ)	Linear Regression (R^2)	Pearson (r)	Spearman (ρ)	***p*-Value**
Fasting blood glucose (mg/dL) (median ± IQR)	0.023	-0.014	-0.012	0.015	0.011	0.012	0.015	0.018	0.017	>0.05
Glycated hemoglobin (%) (median ± IQR)	0.019	0.022	0.017	0.021	0.019	-0.032	0.092	0.089	-0.029	>0.05
Total cholesterol (mg/dL) (median ± IQR)	0.014	-0.009	-0.009	0.018	0.016	0.089	0.078	-0.067	0.089	>0.05
LDL cholesterol (mg/dL) (mean ± SD)	0.031	-0.017	-0.015	0.024	0.022	0.056	-0.019	-0.022	-0.034	>0.05
HDL cholesterol (mg/dL) (median ± IQR)	0.012	0.011	0.011	0.019	0.018	0.073	0.056	-0.034	0.082	>0.05
Serum triglycerides (mg/dL) (median ± IQR)	0.027	-0.025	-0.020	0.025	0.024	-0.048	0.121	0.076	-0.047	>0.05
Body weight (kg) (median ± IQR)	0.018	0.018	0.008	0.022	0.020	0.178	0.099	-0.012	0.169	>0.05
BMI (kg/m^2) (median ± IQR)	0.022	-0.012	-0.014	0.017	0.014	0.092	0.143	0.058	0.098	>0.05
Waist circumference (cm) (mean ± SD)	0.015	0.007	0.005	0.020	0.017	0.045	0.067	-0.024	0.053	>0.05
Systolic blood pressure (mmHg) (median ± IQR)	0.020	-0.019	-0.011	0.014	0.012	-0.067	0.132	0.097	-0.058	>0.05
Diastolic blood pressure (mmHg) (median ± IQR)	0.025	0.015	0.016	0.023	0.021	0.132	0.147	-0.041	0.133	>0.05
Heart rate (bpm) (median ± IQR)	0.017	-0.010	-0.007	0.016	0.015	0.083	0.097	0.063	0.092	>0.05
Sleep duration (h)	N/A	N/A	N/A	0.014	0.235	0.081	0.061	-0.038	0.081	>0.05

Coefficient of Determination, (R^2), ranges from 0 to 1. When (R^2) = 1, the model explains 100% of the variation in the data. Conversely, when (R^2) = 0, the model does not explain any of the variation in the data. Pearson Correlation (r): A value indicating the strength and direction of the association between two variables. The value of r represents the correlation coefficient, which ranges between -1 and 1. A value of $r = 1$ indicates a perfect positive correlation, $r = -1$ indicates a perfect negative correlation, and $r = 0$ indicates no correlation. Spearman correlation coefficient, (ρ), measures the strength and direction of association between two ranked variables. It ranges from -1 to 1, where 1 indicates a perfect positive correlation, -1 indicates a perfect negative correlation, and 0 indicates no correlation.

**Table S6. Linear Regression, Pearson Correlation, and Spearman (health parameters vs. sleep and coffee) obtained during 12th month study - Women**	**Sleep duration**			**Sleep awakens**			**Cup of coffee**			
	Linear Regression (R^2)	Pearson (r)	Spearman (ρ)	Linear Regression (R^2)	Pearson (r)	Spearman (ρ)	Linear Regression (R^2)	Pearson (r)	Spearman (ρ)	***p-Value***
Fasting blood glucose (mg/dL) (median ± IQR)	0.073	-0.102	0.154	0.289	0.092	0.135	0.146	-0.063	0.163	>0.05
Glycated hemoglobin (%) (median ± IQR)	0.125	0.184	-0.040	0.233	0.101	-0.038	0.119	0.042	-0.045	>0.05
Total cholesterol (mg/dL) (median ± IQR)	0.089	-0.050	0.102	0.189	0.067	0.115	0.101	-0.091	0.118	>0.05
LDL cholesterol (mg/dL) (mean ± SD)	0.199	0.098	0.073	0.151	-0.089	0.085	0.169	0.056	0.068	>0.05
HDL cholesterol (mg/dL) (median ± IQR)	0.054	-0.077	0.089	0.317	0.039	0.095	0.073	-0.051	0.094	>0.05
Serum triglycerides (mg/dL) (median ± IQR)	0.142	0.091	-0.050	0.357	-0.044	-0.060	0.138	0.092	-0.055	>0.05
Body weight (kg) (median ± IQR)	0.165	0.057	0.199	0.198	-0.078	0.187	0.175	0.071	0.204	>0.05
BMI (kg/m^2) (median ± IQR)	0.072	-0.030	0.098	0.282	0.112	0.110	0.085	-0.031	0.105	>0.05
Waist circumference (cm) (mean ± SD)	0.072	-0.030	0.054	0.282	0.112	0.065	0.085	-0.031	0.059	>0.05
Systolic blood pressure (mmHg) (median ± IQR)	0.128	0.121	-0.077	0.160	-0.062	-0.070	0.144	0.114	-0.080	>0.05
Diastolic blood pressure (mmHg) (median ± IQR)	0.154	-0.040	0.142	0.227	0.089	0.145	0.158	-0.055	0.150	>0.05
Heart rate (bpm) (median ± IQR)	0.102	0.073	0.091	0.266	-0.051	0.105	0.108	0.076	0.098	>0.05
Sleep duration (h)	N/A	N/A	N/A	0.317	0.039	0.095	0.534*	-0.731 (p=0.037) *	-0.074*	>0.05

Coefficient of Determination, (R^2), ranges from 0 to 1. When (R^2) = 1, the model explains 100% of the variation in the data. Conversely, when (R^2) = 0, the model does not explain any of the variation in the data. Pearson Correlation (r): A value indicating the strength and direction of the association between two variables. The value of r represents the correlation coefficient, which ranges between -1 and 1. A value of $r = 1$ indicates a perfect positive correlation, $r = -1$ indicates a perfect negative correlation, and $r = 0$ indicates no correlation. Spearman correlation coefficient, (ρ), measures the strength and direction of association between two ranked variables. It ranges from -1 to 1, where 1 indicates a perfect positive correlation, -1 indicates a perfect negative correlation, and 0 indicates no correlation.

**Table S7. Linear Regression, Pearson Correlation, and Spearman (health parameters vs. sleep and coffee) obtained during 12th month study - Man**	**Sleep duration**			**Sleep awakens**			**Cup of coffee**			
	Linear Regression (R^2)	Pearson (r)	Spearman (ρ)	Linear Regression (R^2)	Pearson (r)	Spearman (ρ)	Linear Regression (R^2)	Pearson (r)	Spearman (ρ)	***p-Value***
Fasting blood glucose (mg/dL) (median ± IQR)	0.059	-0.087	0.137	0.127	0.074	0.121	0.129	-0.047	0.147	>0.05
Glycated hemoglobin (%) (median ± IQR)	0.112	0.163	-0.028	0.198	0.095	-0.029	0.104	0.035	-0.032	>0.05
Total cholesterol (mg/dL) (median ± IQR)	0.076	-0.038	0.089	0.149	0.058	0.098	0.089	-0.079	0.102	>0.05
LDL cholesterol (mg/dL) (mean ± SD)	0.182	0.081	0.061	0.113	-0.072	0.072	0.152	0.048	0.053	>0.05
HDL cholesterol (mg/dL) (median ± IQR)	0.043	-0.059	0.076	0.278	0.029	0.081	0.061	-0.040	0.081	>0.05
Serum triglycerides (mg/dL) (median ± IQR)	0.128	0.073	-0.038	0.312	-0.036	-0.049	0.125	0.081	-0.043	>0.05
Body weight (kg) (median ± IQR)	0.149	0.045	0.182	0.171	-0.065	0.169	0.158	0.060	0.189	>0.05
BMI (kg/m^2) (median ± IQR)	0.061	-0.022	0.085	0.249	0.097	0.097	0.072	-0.026	0.092	>0.05
Waist circumference (cm) (mean ± SD)	0.058	-0.019	0.043	0.251	0.098	0.053	0.072	-0.022	0.048	>0.05
Systolic blood pressure (mmHg) (median ± IQR)	0.114	0.104	-0.065	0.134	-0.049	-0.058	0.131	0.099	-0.067	>0.05
Diastolic blood pressure (mmHg) (median ± IQR)	0.137	-0.029	0.128	0.195	0.073	0.133	0.142	-0.043	0.132	>0.05
Heart rate (bpm) (median ± IQR)	0.089	0.058	0.078	0.234	-0.040	0.092	0.095	0.065	0.083	>0.05
Sleep duration (h)	N/A	N/A	N/A	0.289	0.031	0.081	0.417	-0.723	-0.713	>0.05

Coefficient of Determination, (R^2), ranges from 0 to 1. When (R^2) = 1, the model explains 100% of the variation in the data. Conversely, when (R^2) = 0, the model does not explain any of the variation in the data. Pearson Correlation (r): A value indicating the strength and direction of the association between two variables. The value of r represents the correlation coefficient, which ranges between -1 and 1. A value of $r = 1$ indicates a perfect positive correlation, $r = -1$ indicates a perfect negative correlation, and $r = 0$ indicates no correlation. Spearman correlation coefficient, (ρ), measures the strength and direction of association between two ranked variables. It ranges from -1 to 1, where 1 indicates a perfect positive correlation, -1 indicates a perfect negative correlation, and 0 indicates no correlation.

Supplementary Materials - Figures

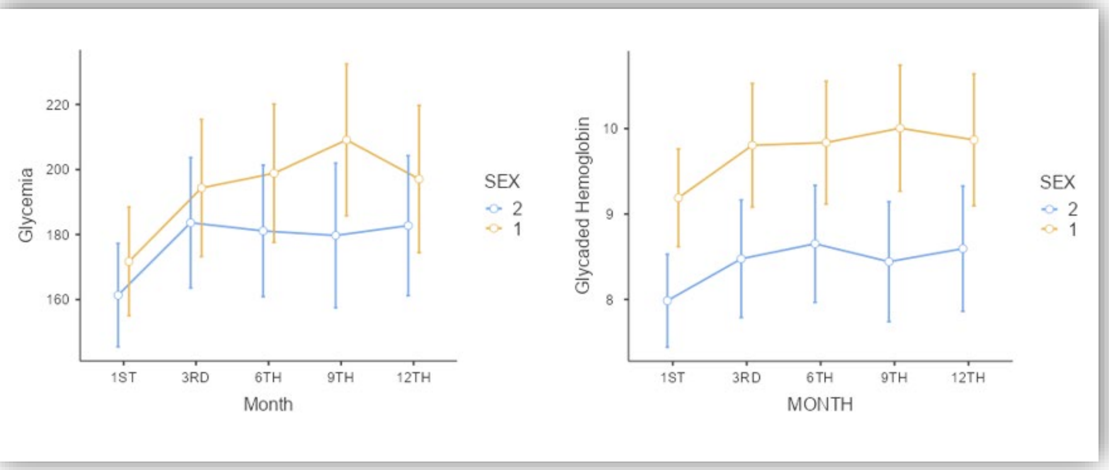

Figure S1. Fasting Glycemia (mg/dL) and Glycated Hemoglobin (%) over 12 months. Non-normal data expressed as median ± interquartile range (IQR). Confidence intervals that do not intersect represent significant differences (Friedman test followed by Durbin-Conover post hoc test for non-parametric data); Sex: 1- Men, 2- Women; $*p < 0.05$.

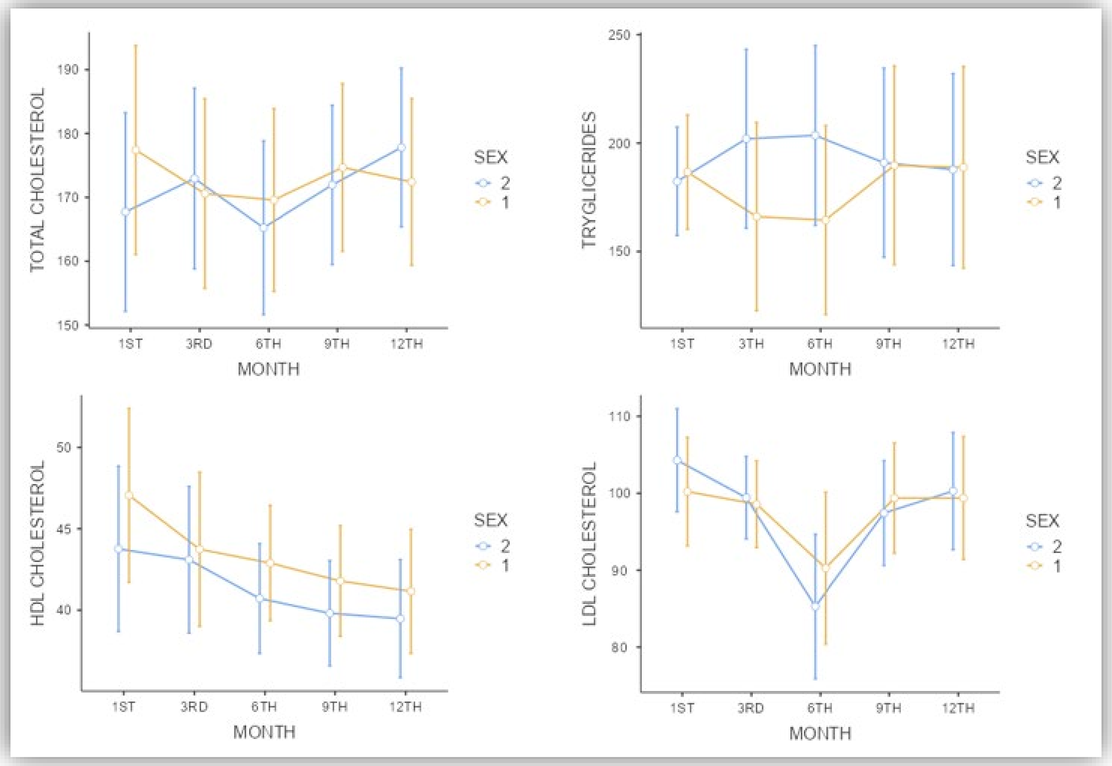

Figure S2. Total cholesterol (mg/dL), LDL cholesterol (mg/dL), HDL cholesterol (mg/dL), and Triglycerides (mg/dL) over 12 months. Non-normal data expressed as median ± interquartile range (IQR) (Total cholesterol and HDL-C); Normal data expressed as mean ± standard deviation (SD) (LDL cholesterol). Confidence intervals that do not intersect represent significant differences (Friedman test followed by Durbin-Conover post hoc test for non-parametric data; repeated measures ANOVA followed by Tukey post hoc test for parametric data); Sex: 1- Men, 2- Women; *p < 0.05.

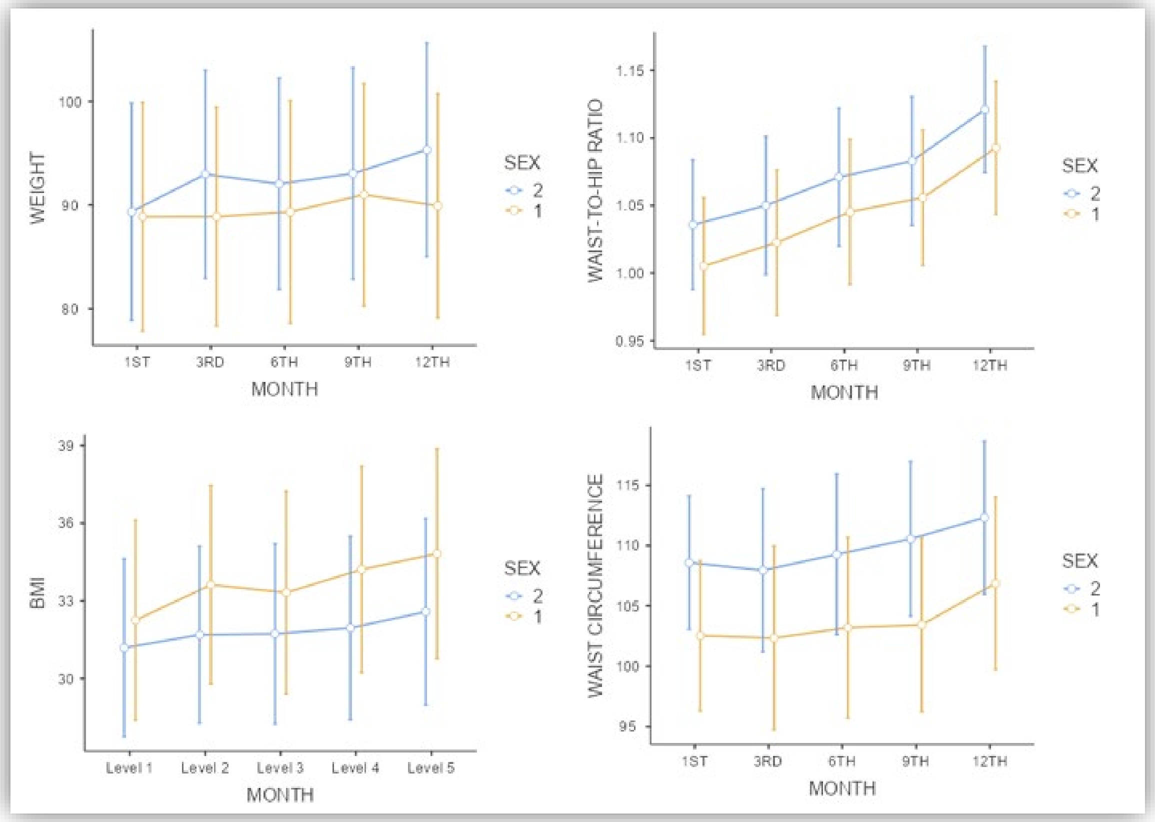

Figure S3. Weight (kg), Body Mass Index (BMI) (kg/m^2), Waist Circumference (cm), and Waist-Hip Ratio (WHR) (unit) over 12 months. Non-normal data expressed as median ± interquartile range (IQR) (Weight, BMI, and WHR); Normal data expressed as mean ± standard deviation (SD) (Waist Circumference). Confidence intervals that do not intersect represent significant differences (Friedman test followed by Durbin-Conover post hoc test for non-parametric data; repeated measures ANOVA followed by Tukey post hoc test for parametric data); Sex: 1- Men, 2- Women; *$p < 0.05$.

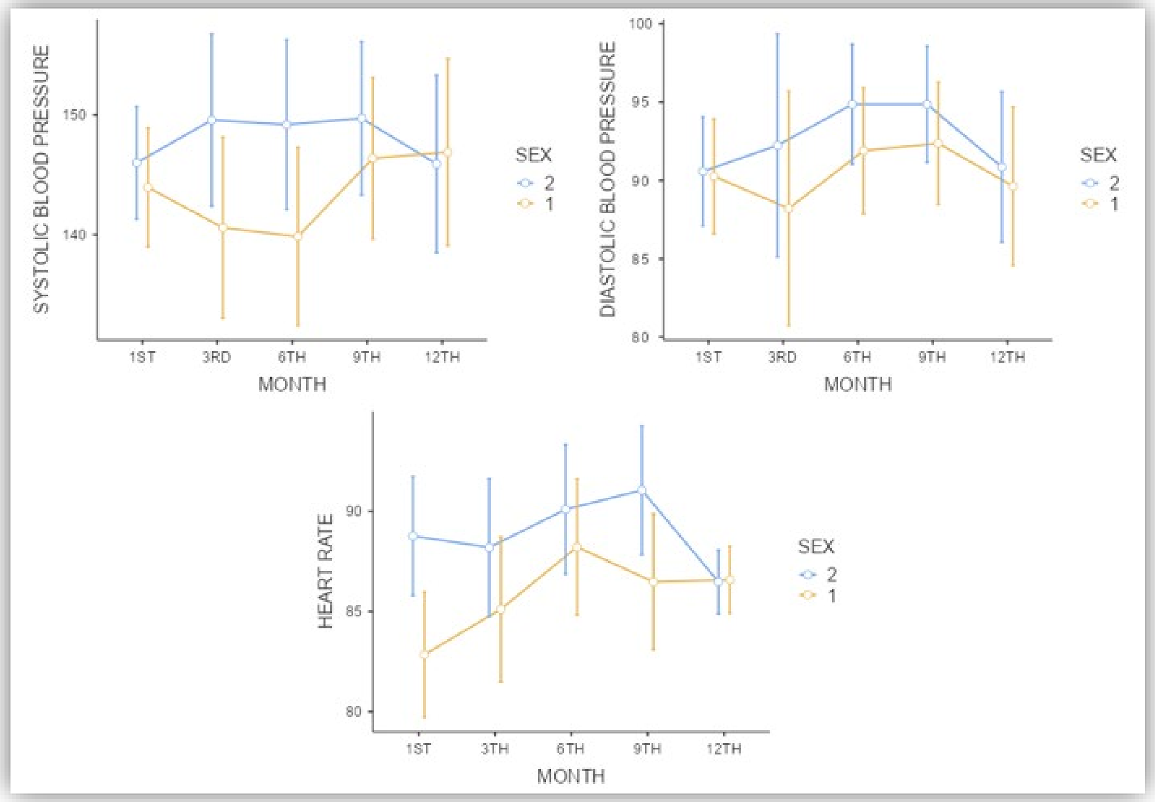

Figure S4. Blood Pressure (mmHg) and Heart Rate (bpm) over 12 months. Non-normal data expressed as median ± interquartile range (IQR) (Systolic Blood Pressure and Heart Rate); Normal data expressed as mean ± standard deviation (SD) (Diastolic Blood Pressure). Confidence intervals that do not intersect represent significant differences (Friedman test followed by Durbin-Conover post hoc test for non-parametric data; repeated measures ANOVA followed by Tukey post hoc test for parametric data); Sex: 1- Men, 2- Women; $*p < 0.05$.

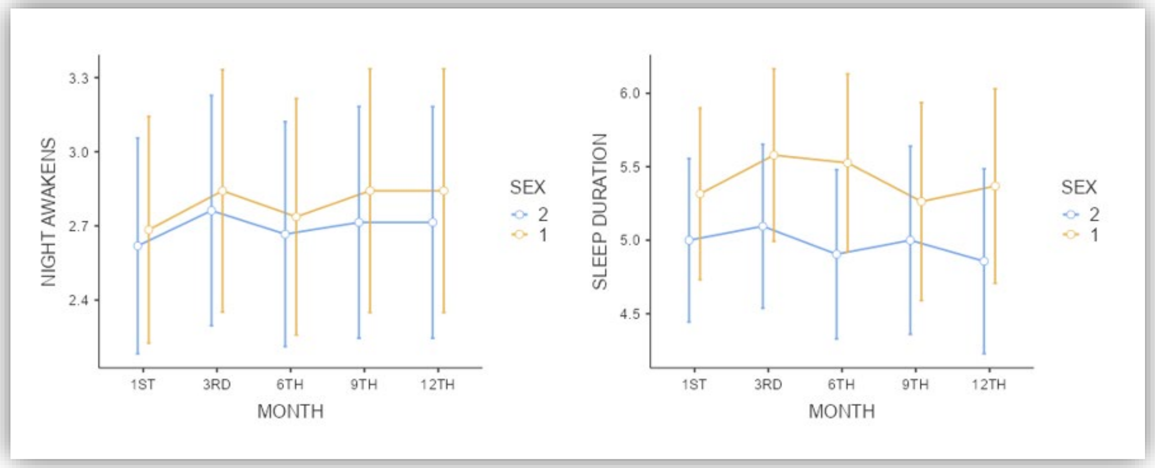

Figure S5. Night Awakens (unit) and Sleep Duration (h) over 12 months. Non-normal data expressed as median ± interquartile range (IQR) (Night Awakens); Normal data expressed as mean ± standard deviation (SD) (Sleep Duration). Confidence intervals that do not intersect represent significant differences (Friedman test followed by Dunn's post hoc test for non-parametric data; repeated measures ANOVA followed by Tukey post hoc test for parametric data); Sex: 1- Men, 2- Women; *$p < 0.05$.
